# Annual recurrence and period-composite persistence of child–heat priority across six contrasting urban systems

**DOI:** 10.3389/fpubh.2026.1984653

**Published:** 2026-10-20

**Authors:** Xin Bian, Shufang Sun

**Affiliations:** 1 School of Art and Design, Shandong Women’s University, Jinan, China; 2 Wellington School of Architecture, Victoria University of Wellington, Wellington, New Zealand; 3 ZhongQi MaiHe Tianjin, Engineering Design and Research Institute Co., Ltd., Tianjin, China

**Keywords:** children, exposure inequality, heat adaptation, land-surface temperature, urban heat

## Abstract

Urban heat adaptation requires spatial evidence showing where larger child populations coincide with high surface heat, especially where comparable paediatric health records are unavailable. Single-year hotspot maps cannot determine whether priority areas recur annually, persist across multi-year composites or remain reproducible across thermal products. A harmonised 10-year ecological framework was applied to New York, London, Singapore, Nairobi, São Paulo and Brisbane using annual WorldPop estimates for children aged 0–14 years and Landsat warm-season land-surface temperature on a common 1-km hexagonal grid from 2016 to 2025. Within each city-year, child count and heat anomaly were converted to percentile ranks and combined using an equal-weight geometric mean. Cells at or above the 90th score percentile formed the annual priority set. Three continuous periods defined period-composite trajectories, while annual membership quantified recurrence, consecutive runs, transitions, entries and exits. The panel contained 78,300 grid-year observations across 60 city-years. Annual priority sets contained 23.3–44.8% of eligible children across cities and 3.1–30.9 percentage points more children than heat-only sets. Among 10-year common-panel cells, agreement between period-composite persistence and annual membership in at least 8 years was moderate to strong but incomplete (Jaccard = 0.55–0.71). Landsat and MODIS/Terra thermal ranks were positively associated (*ρ* = 0.35–0.86), whereas annual upper-decile overlap was only 0.11–0.38. Child concentration, annual recurrence, period-composite persistence and boundary reproducibility therefore represent distinct planning dimensions. The framework supports first-stage prioritisation and local verification but does not estimate individual health risk or causal effects.

## 1 Introduction

Children are especially sensitive to urban heat because immature thermoregulation, dependence on caregivers and time spent in schools or outdoor settings can restrict behavioural adaptation (1). High temperature has been associated with paediatric respiratory, renal, electrolyte and febrile morbidity, although the magnitude and timing of associations vary by age, diagnosis and exposure definition (2). A recent scoping review found that this evidence remains geographically concentrated and methodologically heterogeneous (3). For urban adaptation, the resulting challenge is not only to establish that heat affects children, but also to identify where child-focused intervention should be prioritised within cities.

Neighbourhood heat is redistributed by vegetation, built form, surface materials and urban morphology, making exposure spatially uneven even within the same climatic setting (4). Small-area heat-vulnerability indices therefore combine environmental and demographic indicators to locate areas of potential concern (5–7). However, indicator selection, weighting and spatial units vary substantially among indices, which limits comparison and transferability (8). Recent global and multimodal frameworks improve standardisation and representation of spatial inequality, but they remain predominantly cross-sectional and treat children as one component of a broader sensitivity construct rather than as the explicit longitudinal population of interest (9, 10).

The child–heat literature has consequently developed along two partly separate methodological tracks. Case-crossover, time-series and condition-specific observational studies estimate short-term associations between temperature and paediatric emergency visits, admissions or morbidity while controlling seasonality and delayed effects (11–15). These designs provide stronger outcome inference than a spatial screening index, but they depend on health-service records that are not consistently accessible or comparable across cities. Spatial vulnerability studies are more readily transferable to planning, but they commonly describe one time point and do not test how the location of child-specific priority changes from year to year.

Open satellite and gridded population products create an opportunity to connect these two tracks without claiming individual health risk. Longitudinal heat-equity research has shown that population exposure distributions can change through time (16, 17). Satellite-based epidemiology has also demonstrated the value of spatially resolved temperature fields for population-health analysis (18). Yet the available approaches rarely align annual child population, annual warm-season heat and annual land cover on identical spatial support, and cross-sectional hotspot maps cannot distinguish period-composite persistent, emerging, declining and intermittent planning priorities. The evidence-search strategy, selection flow and structured evidence map supporting this gap are reported in Supplementary Methods S1, Supplementary Figure S1 and Supplementary Tables S1–S4.

A further limitation concerns cross-product agreement and decision stability. Agreement between continuous temperature surfaces does not necessarily imply agreement in the small upper tail selected for intervention, because modest rank differences can change membership of a top-decile priority set. Independent thermal-product comparison and explicit testing of priority-set overlap are therefore needed alongside spatial correlation. Taken together, the unresolved problem is a harmonised, child-specific and outcome-independent framework that can follow priority geographies through time, operate under explicit data-quality rules and distinguish sensor agreement from stability of the selected planning set.

This study addresses that problem using same-year open data for New York, London, Singapore, Nairobi, São Paulo and Brisbane from 2016 to 2025. Annual WorldPop estimates for children aged 0–14 years, Landsat warm-season land-surface temperature and Dynamic World land-cover fractions were matched on a common 1-km hexagonal grid. Within each city-year, child count and heat anomaly were converted to percentiles and combined as a geometric-mean priority score; annual upper-decile sets were then summarised into early, middle and recent periods and evaluated against season-matched MODIS/Terra temperature. The framework is intended for first-stage planning screening and does not estimate individual exposure, clinical risk or causal intervention effects.

The study has three objectives and corresponding contributions.

1. It establishes a reproducible longitudinal workflow that aligns annual child population, surface heat and land-cover context on identical spatial support under explicit eligibility and water-screening rules.
2. It quantifies the concentration of children in the annual upper-decile priority set and distinguishes period-composite persistent, period-specific and intermittent priority trajectories across six contrasting cities.
3. It evaluates both continuous Landsat–MODIS heat-rank agreement and annual upper-decile Jaccard overlap, separating general thermal concordance from stability of the policy-relevant priority classification.

The remainder of the paper is organised as follows. Section 2 describes the study frame, annual data alignment, thermal quality control, priority-score construction and cross-product agreement procedures. Section 3 reports longitudinal coverage, priority concentration, spatial trajectories and thermal-product agreement. Section 4 interprets the findings for urban heat adaptation and states the methodological boundaries. Section 5 summarises the conclusions.

## 2 Data and methods

The analysis followed a seven-stage workflow (Figure 1). Official city boundaries were first converted to a common 1-km hexagonal support. Annual child population, warm-season land-surface temperature (LST) and land-cover observations were then assigned to the same grid identifiers. Thermal and land-coverage criteria were applied before ranking, annual child–heat priority scores were calculated, annual estimates were summarised into three periods, and the resulting patterns were evaluated through trajectory, independent-product agreement and robustness analyses. All longitudinal analyses covered 2016–2025.

**Figure 1:**
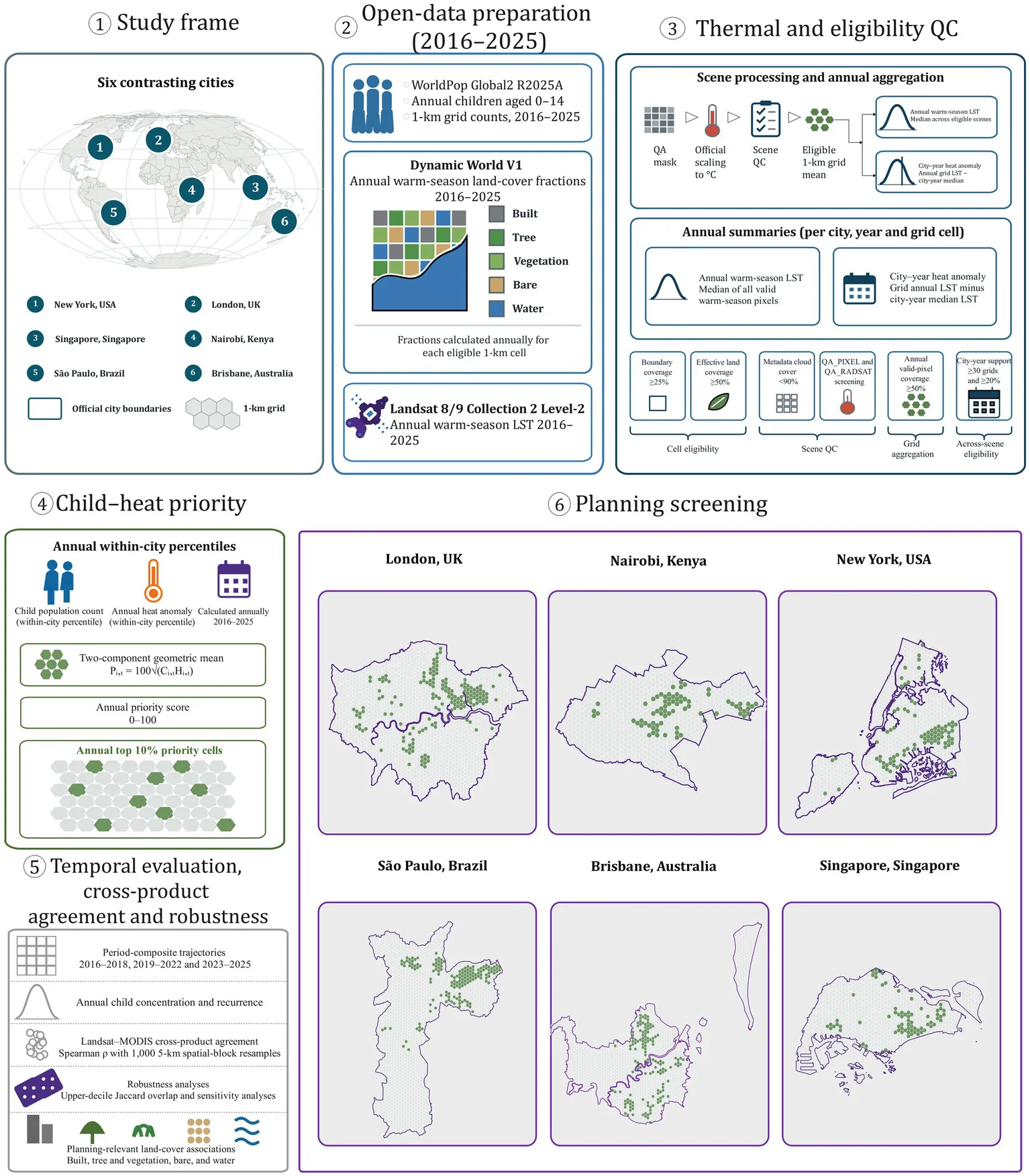
Harmonised workflow for the six-city longitudinal child–heat priority analysis. The workflow links study-frame definition, annual open-data preparation, Landsat thermal processing and eligibility checks, annual priority scoring, longitudinal evaluation and planning screening. Annual data cover 2016–2025 and are summarised into early (2016–2018), middle (2019–2022) and recent (2023–2025) periods. The schematic summarises the study design and analytical order; it does not depict observed results.

The Supplementary material provides the evidence and technical detail needed to audit the main analysis without repeating it. Supplementary Methods S1, Supplementary Figure S1 and Supplementary Tables S1, S4 document the search, screening and structured gap coding underpinning the Introduction. Data products and annual alignment are specified in Supplementary Methods S2 and Supplementary Table S2; the complete grid, scene and city-year quality-control record is reported in Supplementary Methods S3 and Supplementary Table S3; and score construction, period trajectories, spatial inference and uncertainty analyses are detailed in Supplementary Methods S4. These items are cross-referenced at the relevant points in the main text so that data losses, threshold choices and robustness judgements remain traceable. The remaining analytical results and robustness checks are reported in Supplementary Tables S5–S21, which are listed in numerical order in the Supplementary material inventory.

### 2.1 Study areas and common spatial support

A longitudinal comparative ecological design was applied to New York, London, Singapore, Nairobi, São Paulo and Brisbane. The cities were selected by purposive maximum variation across continents, hemispheres, climatic settings, coastal form and development context, while inclusion required an official municipal boundary and annual observations from the selected open-data products. The six cities are therefore contrasting cases used to test workflow transferability, not a probability sample of global cities.

Boundary polygons were repaired, dissolved and projected to locally appropriate metric coordinate systems before nominal 1-km hexagons were generated. A boundary hexagon was retained when at least 25% of its area fell within the city. The same city and grid identifiers were used for population aggregation, LST estimation, land-cover characterisation, period construction and cross-product comparison. Effective land coverage, thermal coverage and observation counts were stored for each grid-year so that changes in spatial support were not interpreted as temporal change.

### 2.2 Data sources, warm seasons and analysis periods

Four public products were used. WorldPop Global2 R2025A provided annual gridded population estimates by age and sex. Annual child population was defined as the summed WorldPop estimates for ages 0–14 within each grid-year. This broad age band provided a consistent measure of the child population potentially served by place-based planning across all cities and study years. Landsat 8 and 9 Collection 2 Level-2 provided 30-m surface-temperature observations. Dynamic World V1 supplied 10-m land-cover class probabilities, and MODIS/Terra MOD11A1 V061 supplied daily 1-km daytime land-surface temperature for independent thermal comparison. All products were reduced to the common hexagonal support following product-specific quality control. Product versions, temporal coverage and analytical roles are documented in Supplementary Methods S2 and Supplementary Table S2.

City-specific three-month windows were applied consistently across all study years: June–August for New York and London, April–June for Singapore, January–March for Nairobi, and December–February for São Paulo and Brisbane. These windows represent locally warm or climatologically appropriate periods identified from the cited 1991–2020 station or national climatologies. Singapore has limited annual temperature seasonality; its April–June window encompasses the annual temperature peak in the official 1991–2020 24-h monthly temperature normals. The climatological rationale, monthly evidence and institutional source for each city are provided in Supplementary Table S17.

A fixed three-month window was applied in each year to maintain consistent temporal sampling of Landsat observations within cities. The windows were defined separately according to each city’s climatic setting and therefore differed across temperate, subtropical, tropical-highland and equatorial environments. This design supported year-to-year comparison within cities while accommodating regional differences in seasonality. For São Paulo and Brisbane, the December–February window was assigned to the year in which December occurred and extended through February of the following calendar year.

For New York, London, Nairobi, São Paulo and Brisbane, the selected windows corresponded to the locally warmest or broadly hottest contiguous three-month periods identified from the cited climatologies. Singapore was assessed separately because its weak annual temperature seasonality allows more than one adjacent three-month window to represent conditions near the annual temperature maximum. Seasonal-window sensitivity was evaluated by comparing the April–June primary window with a March–May pre-southwest-monsoon window. Agreement between the two specifications was assessed using annual LST-rank correlations, priority-percentile correlations, annual upper-decile Jaccard overlap and overlap between the period-composite persistent sets. The corresponding results are reported in the Results and Supplementary Tables S18, S19.

Annual analyses covered 2016–2025. For temporal interpretation, annual estimates were additionally summarised into three contiguous descriptive periods: early (2016–2018), middle (2019–2022) and recent (2023–2025). The 3–4–3 partition provided near-comparable coverage of the beginning, middle and end of the available decade, and its boundaries were specified independently of observed temporal change points. Sensitivity to boundary placement was assessed using adjacent 3–3–4 and 4–3–3 partitions, with agreement evaluated by Jaccard overlap between the resulting period-composite persistent sets. The corresponding results are reported in the Results and Supplementary Table S20. Missing annual population or thermal values were not interpolated.

### 2.3 Landsat LST processing and eligibility

Landsat images were filtered by city boundary, year, predefined warm-season window, processing level and metadata cloud cover below 90% in Google Earth Engine. Fill, dilated cloud, cirrus, cloud, cloud shadow and snow were removed with QA_PIXEL flags, and radiometrically saturated pixels were removed with QA_RADSAT. Surface temperature was obtained from ST_B10_ using the Collection 2 scale and offset, LST (°C) = ST_B10_ × 0.00341802 + 149.0–273.15. The annual grid-level LST was the median of all valid warm-season pixels. The number of contributing images, the mean valid-observation count and the annual valid-pixel fraction were retained as audit variables.

To emphasise within-city spatial contrast rather than between-city climatic differences, annual heat anomaly was calculated relative to the median LST of eligible cells in the same city-year as defined in Equation 1:

$$A_{\mathrm{it}}=\mathrm{LS}T_{\mathrm{it}}-\text{media}n_{j}(\mathrm{LS}T_{\mathrm{jt}})$$ (1)

Where LST*_it_* is the annual warm-season LST for grid *i* in year *t* and the median is taken over eligible grids *j* in the same city-year. Positive values identify cells warmer than the contemporaneous city-year median; the measure is a relative surface-heat anomaly and is not equivalent to canopy-layer air temperature or an urban–rural heat-island intensity. Subtracting a common city-year median does not alter the within-city rank ordering of LST. The anomaly was retained to express each grid’s thermal position relative to its contemporaneous urban surface environment, not to change the heat percentile or priority classification.

Eligibility was evaluated before percentile ranking. A boundary hexagon required at least 25% city coverage and at least 50% effective land coverage. For the primary annual analysis, a grid-year required a non-missing child count and annual LST, at least 50% valid Landsat pixel coverage after pixel-level quality masking, and non-water status. A city-year was retained only when at least 30 complete child–heat grids remained and these represented at least 20% of the city’s included longitudinal grid support. Period composites used the archived 20% annual heat-validity rule and required at least one eligible thermal year within the period. A cell was classified as water dominated when its median annual water fraction within the period was at least 50%. Water cells were excluded from ranking and displayed separately so that coastal water did not create artificial cool zones or inflate apparent completeness (Supplementary Methods S3; Supplementary Table S3).

### 2.4 Annual child–heat priority score

Within each eligible city-year, child count and heat anomaly were independently converted to percentile ranks on a 0–1 scale. These ranks are denoted *C_it_* and *H_it_*, respectively, for grid *i* in year *t*. Average ranks were assigned to tied observations. Percentiles were calculated separately within each city and year, so the score represents relative local priority rather than an absolute comparison of population or temperature among cities. The annual child-heat priority score was calculated as Equation 2:

$$P_{\mathrm{it}}=100\times \sqrt{C_{\mathrm{it}}\;H_{\mathrm{it}}}$$ (2)

The geometric mean was selected as the primary planning function because it limits compensation between the child-population and heat dimensions, but it was not assumed to be the uniquely optimal aggregation rule. Equal weighting reflects the normative judgement that both dimensions are necessary and equally important for first stage planning screening. Percentile transformation placed the two components on a common scale and reduced dependence on their different marginal distributions. Under the implemented rank/n convention, all percentile ranks were strictly positive, thereby avoiding undefined geometric means at zero. Average ranks were assigned to ties, and all cells at or above the 90th score percentile were retained. Consequently, ties at the threshold could produce a priority set marginally larger than exactly 10% of eligible cells. The upper-decile threshold was used as a transparent planning rule rather than as an estimated biological or health-risk threshold. Sensitivity analyses evaluated the principal normative choices underlying the index by comparing child count with child density and population share, the rank/n convention with a zero-based percentile convention, and the geometric mean with arithmetic, harmonic and minimum-component aggregation. Additional analyses examined unequal component weights, relative heat anomaly versus raw land-surface-temperature rank, and priority thresholds of 5%, 10 and 20%. Detailed specifications and results are reported in Supplementary Tables S9, S10, S16. Full scoring, trajectory and uncertainty procedures are provided in Supplementary Methods S4.

Child concentration in the annual priority set was calculated as using Equation 3:

$$K_{\mathrm{ct}}=100\times \frac{\sum_{i\in T_{\mathrm{ct}}}N_{\mathrm{it}}}{\sum_{i\in E_{\mathrm{ct}}}N_{\mathrm{it}}}$$ (3)

Where *T_ct_* is the annual upper-decile priority set for city *c* in year *t*, *E_ct_* is the corresponding set of eligible grids, and *N_it_* is the number of children in grid *i*. A value of 10% is reported only as the descriptive share expected if children were distributed uniformly across equal-area eligible grids; it was not treated as a statistical null hypothesis. To distinguish concentration arising from the joint child-heat index from that produced by alternative selection rules, the observed share was compared with 10,000 randomly selected sets containing the same number of eligible grids and with the upper decile defined by heat rank alone.

### 2.5 Period composites and trajectory classification

For each early, middle and recent period, a grid was included when at least one annual estimate was eligible. Median child count and median heat anomaly were calculated across the eligible years in that period; both components were then reranked and the geometric priority score was recalculated within each city-period. This procedure prevents annual missingness from being filled by interpolation and makes every period map a relative within-city comparison.

Priority change was evaluated at two temporal resolutions. At the period level, percentile-point changes and upper-decile membership were compared across the early, middle and recent composites. Cells were classified as never selected, early only, middle only, recent only, period-composite persistent (selected in all three periods), or following another intermittent trajectory. Annual recurrence was characterised by each grid using the number and proportion of eligible years in the annual upper decile, the longest consecutive run of membership, transition frequency, entry and exit counts, and the mean annual priority percentile. A run was interrupted by either non-membership or an ineligible year. Transitions were evaluated only between consecutive eligible years, with an entry defined as a change from non-membership to membership and an exit as the reverse. The correspondence between annual recurrence and period-composite persistence was evaluated among common-panel grids that were eligible in all 10 annual panels and all three period composites. Spearman’s rank correlation was used to assess the association between period-composite persistence and the proportion of years in the annual upper decile. For set-based comparisons, high annual recurrence was defined as membership in at least eight of the 10 annual priority sets. Its agreement with period-composite persistence was quantified using the Jaccard index and Cohen’s κ. Membership in all 10 annual priority sets was examined as a stricter sensitivity definition. Water-dominated grids were displayed in pale blue, whereas land grids without an eligible estimate throughout a given period were displayed in grey. These measures describe the recurrence and spatial redistribution of planning priority rather than uninterrupted heat exposure or individual exposure histories.

### 2.6 Cross-product agreement, uncertainty, robustness and software

Landsat heat percentiles were compared with season-matched MODIS/Terra MOD11A1 V061 daytime LST percentiles as an assessment of cross-product agreement. Continuous agreement was quantified with Spearman rank correlation. Ninety-five per cent confidence intervals were obtained from 1,000 resamples of 5-km spatial blocks; each sampled block carried all available years, thereby preserving the repeated temporal series within location while reducing the influence of local spatial dependence. City-year correlations were additionally summarised as a temporal sensitivity analysis. Decision-set agreement was evaluated separately using the annual Jaccard overlap defined in Equation 4 between the Lands\at and MODIS upper-decile heat sets:

$$J_{\mathrm{ct}}=\frac{|L_{\mathrm{ct}}\cap M_{\mathrm{ct}}|}{|L_{\mathrm{ct}}\cup M_{\mathrm{ct}}|}\;$$ (4)

Where *L_ct_* and *M_ct_* denote the sets of cells classified in the upper decile of the heat rankings derived from Landsat and MODIS, respectively, for city *c* in year *t*. Reporting both Spearman’s rank correlation and Jaccard overlap distinguishes broad agreement in thermal rankings from agreement in the exact set of cells selected at the upper-decile threshold.

The city-year audit recorded total boundary cells, common-panel cells, annual eligible coverage, water-dominated cells, contributing Landsat images, valid-observation density, complete child–heat grids, MODIS matches and the eligible child-population denominator. Robustness analyses compared upper 5%, 10 and 20% priority thresholds; child count, population share and clipped-cell density; one-based and zero-based percentile conventions; raw LST and relative heat anomaly; arithmetic, harmonic, minimum-component and child- or heat-weighted score formulations; and 20%, 50 and 70% valid-coverage rules. Leave-one-city-out summaries assessed whether the pooled land-cover–heat association was dominated by any single city. Leave-one-year-out summaries and a common 10-year support additionally assessed temporal and spatial-sample stability. Seasonal sensitivity compared Singapore’s April–June primary window with the original March–May window, and alternative 3–3–4 and 4–3–3 period partitions tested dependence on the primary cut-points. No causal effect, clinical outcome or individual health risk was estimated.

Remote-sensing extraction was implemented in Google Earth Engine. Reproducible data management, statistical analysis and document assembly used Python 3.12.13 with pandas 3.0.1, NumPy 2.3.5 and python-docx 1.2.0. Spatial processing used GeoPandas, rasterio, shapely and pyproj in the archived analysis environment. Exact dependency locks, Earth Engine scripts, analysis code and figure-source tables will accompany the data release.

## 3 Results

This section evaluates child–heat priority across the six cities from 2016 to 2025. It first establishes whether the harmonised workflow produced adequate and comparable analytical coverage and quantifies the share of children located in the annual upper-decile priority set (Section 3.1). It then examines annual and period-scale changes in the concentration of children within priority areas (Section 3.2). The component and transition maps are subsequently used to determine whether temporal differences reflect stable spatial cores, local rank changes or intermittent upper-decile membership (Section 3.3). Finally, Landsat thermal rankings are compared independently with season-matched MODIS observations, and vegetation–heat gradients are reported as planning context (Section 3.4).

### 3.1 Analytical coverage and concentration of children in annual priority areas

The annual analytical panel contained 78,300 grid-year records across all 60 city-years (Table 1). Annual complete-grid counts were invariant in New York (1,004) and Nairobi (812) and varied only slightly in London (1,719–1,730). Larger ranges occurred in Singapore (862–946), São Paulo (1,261–1,703) and Brisbane (769–1,583). The median percentage of boundary cells meeting the analytical eligibility criteria was 100.0% in New York and London, 99.8% in Nairobi, 99.9% in São Paulo and Brisbane, and 91.0% in Singapore. The number of cells represented in all 10 annual panels ranged from 498 in Brisbane to 1,718 in London. São Paulo’s unusually low median LST in 2020 (20.6 °C) coincided with its smallest complete-grid set (1,261 cells), eight contributing Landsat images and the lowest median number of valid observations per grid (1.07). Restricting all years to the same 1,213 10-year common-panel cells did not remove the anomaly: the 2020 median was 19.7 °C, compared with 30.4–35.4 °C in the remaining years on the same spatial support. The wide annual range was therefore driven primarily by 2020 but was not explained by changes in spatial support alone. Excluding 2020 narrowed the complete-grid annual median range from 20.6–38.7 °C to 33.9–38.7 °C. Because image and observation support was comparatively sparse in 2020, the estimate was retained but interpreted cautiously rather than treated as unambiguous evidence of climatic change. The full annual audit is reported in Supplementary Table S7.

**Table 1:** Annual analytical coverage, child concentration, spatial support, image availability and population denominators across the six study settings.

Panel A. Annual analytical coverage and child concentration				
City	Complete grids per year	Mean children in priority set (%)	Annual range (%)	Median annual LST range (°C)
Brisbane	769–1,583	35.0	33.2–35.8	34.1–38.2
London	1,719–1,730	23.3	22.5–24.2	29.5–36.9
Nairobi	812–812	31.2	21.4–38.8	34.0–42.0
New York	1,004–1,004	28.2	26.1–30.5	34.7–39.4
São Paulo	1,261–1,703	24.2	23.5–27.1	20.6–38.7
Singapore	862–946	44.8	35.2–51.6	32.0–38.5

Panel B. Spatial support				
City	Boundary cells	Ten-year common-panel cells	Eligible boundary cells, median (range), %	Water-dominated cells, median
Brisbane	1,583	498	99.9 (48.6–100.0)	97
London	1,730	1,718	100.0 (99.4–100.0)	6
Nairobi	814	812	99.8 (99.8–99.8)	2
New York	1,004	1,004	100.0 (100.0–100.0)	150
São Paulo	1,703	1,213	99.9 (74.0–100.0)	36
Singapore	996	664	91.0 (86.5–95.0)	198

Panel C. Coverage timing, image support and population denominator				
City	Minimum-coverage year(s)	Maximum-coverage year(s)	Median contributing images	Mean annual eligible children
Brisbane	2018	2016, 2021, 2022, 2024, 2025	14.5	209,412
London	2019	2017, 2018, 2020, 2022, 2023, 2024, 2025	9.5	1,685,360
Nairobi	2016–2025	2016–2025	6.0	1,688,640
New York	2016–2025	2016–2025	16.5	1,454,295
São Paulo	2020	2016, 2021, 2023, 2024	11.5	2,142,664
Singapore	2020	2018	5.5	602,732

Complete grids are land grids satisfying the annual eligibility criteria for both the child-population and thermal components. Priority sets comprise cells at or above the within-city-year 90th percentile of the child–heat priority score. Ten-year common-panel cells were eligible in every year from 2016 to 2025. Eligible boundary coverage is the percentage of all boundary cells satisfying the annual analytical criteria. Contributing images refer to Landsat observations retained after the specified quality-control procedures. For Nairobi and New York, minimum and maximum coverage were identical in every study year; these years are therefore reported as 2016–2025. LST, land-surface temperature.

The annual upper-decile priority sets contained an average of 23.3–44.8% of eligible children across the six cities. Mean concentration was highest in Singapore (44.8%; annual range 35.2–51.6%), followed by Brisbane (35.0%; 33.2–35.8%), Nairobi (31.2%; 21.4–38.8%), New York (28.2%; 26.1–30.5%), São Paulo (24.2%; 23.5–27.1%) and London (23.3%; 22.5–24.2%). Random selections containing the same number of cells included approximately 10.0% of eligible children on average, whereas heat-only upper-decile sets contained city means of 7.4–21.1%. The joint child–heat priority sets therefore contained 3.1 percentage points more children than the corresponding heat-only sets in São Paulo and 30.9 percentage points more in Singapore. The difference was positive in every city-year and ranged from 2.2 to 38.8 percentage points. Concentration above 10% was interpreted as a consequence of incorporating child population into the selection rule, while comparison with the heat-only sets quantified the additional population concentration associated with the joint index. Complete city-level and annual reference comparisons are reported in Supplementary Table S6.

### 3.2 Temporal redistribution of child–heat priority

High-priority areas formed contiguous multi-cell patches in all six cities during the early, middle and recent periods, but their extent, connectivity and edge membership changed to different degrees (Figure 2). The main high-priority structures remained comparatively intact in São Paulo and Brisbane; New York and London retained their broad structures with local adjustment around their margins; and redistribution of higher-ranked cells was more apparent in Singapore and Nairobi. Because percentiles were recalculated within each city-period, these differences represent movement in relative priority rather than a uniform rise or fall in absolute citywide heat. All non-water land cells had eligible estimates in all three periods in London, Nairobi, São Paulo and Brisbane. New York contained 154 water-dominated cells, whereas Singapore contained 247 water-dominated cells and 21 non-water land cells without an eligible estimate in at least one period. The mapped transitions therefore do not arise from treating water or missing land as low priority; they show genuine within-city reordering of the joint child and heat surface.

**Figure 2:**
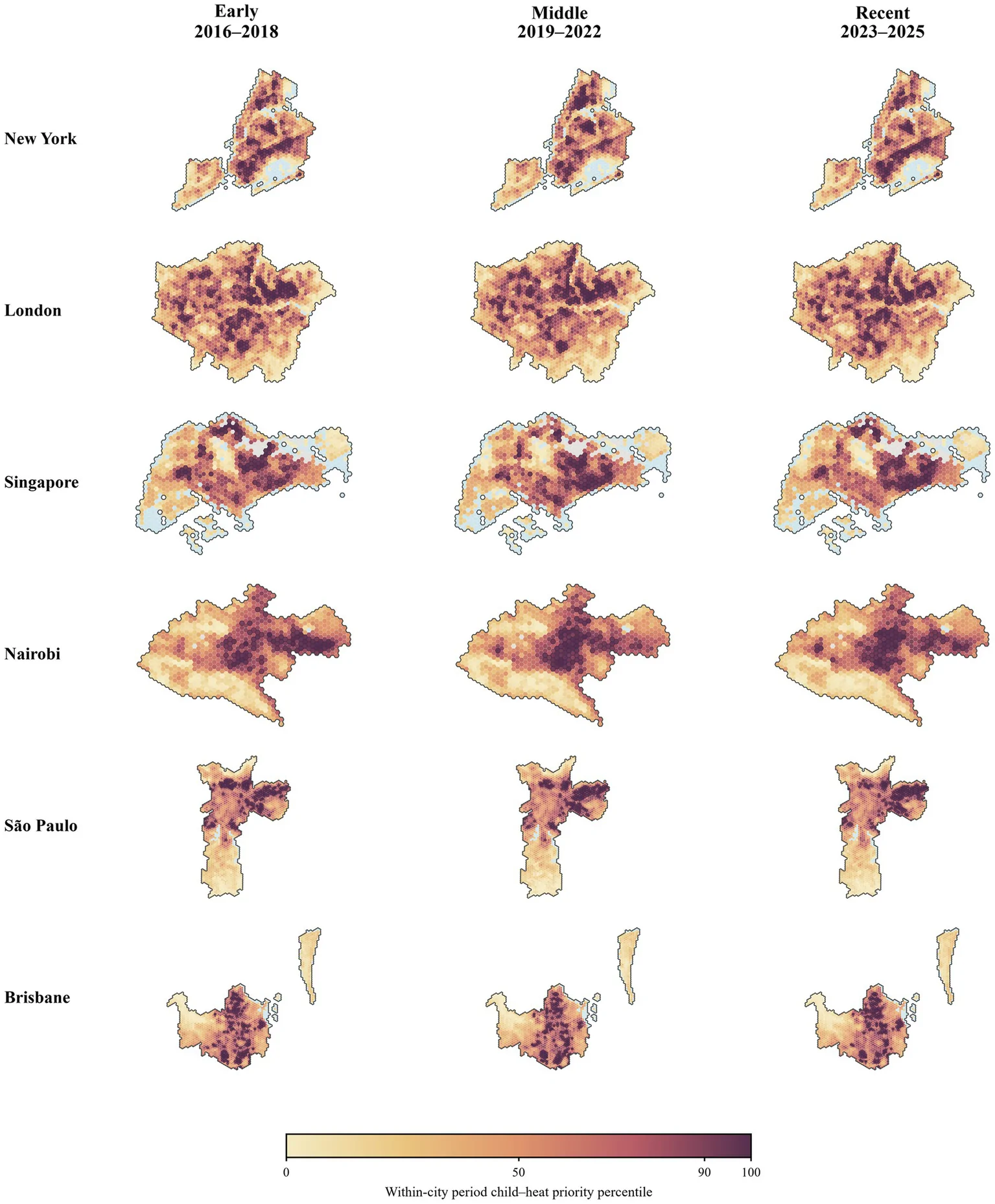
Child–heat priority across the early, middle and recent analysis periods. Columns show the early (2016–2018), middle (2019–2022) and recent (2023–2025) periods; colours represent child–heat priority percentiles within each city-period. Pale blue denotes water-dominated cells with a median annual water fraction ≥50%, and grey denotes non-water land without an eligible period estimate.

High child-population and high warm-season heat-anomaly cells overlapped only partly in every city (Figure 3). Some cells contained more children but ranked lower thermally, while others were relatively hot but contained fewer children; neither group entered the highest combined ranks on the strength of one component alone. The highest child–heat priorities occurred where both percentiles were elevated, so the combined surface is neither a redrawing of the population layer nor a redrawing of the heat layer. Tree and vegetation cover varied within both high- and low-priority areas and was not included in the score. It instead describes differences in planning context among cells with otherwise similar priority. Figure 2 therefore establishes how the priority geography changed between periods, whereas Figure 3 explains how population, heat and land-cover context produced the observed spatial configuration.

**Figure 3:**
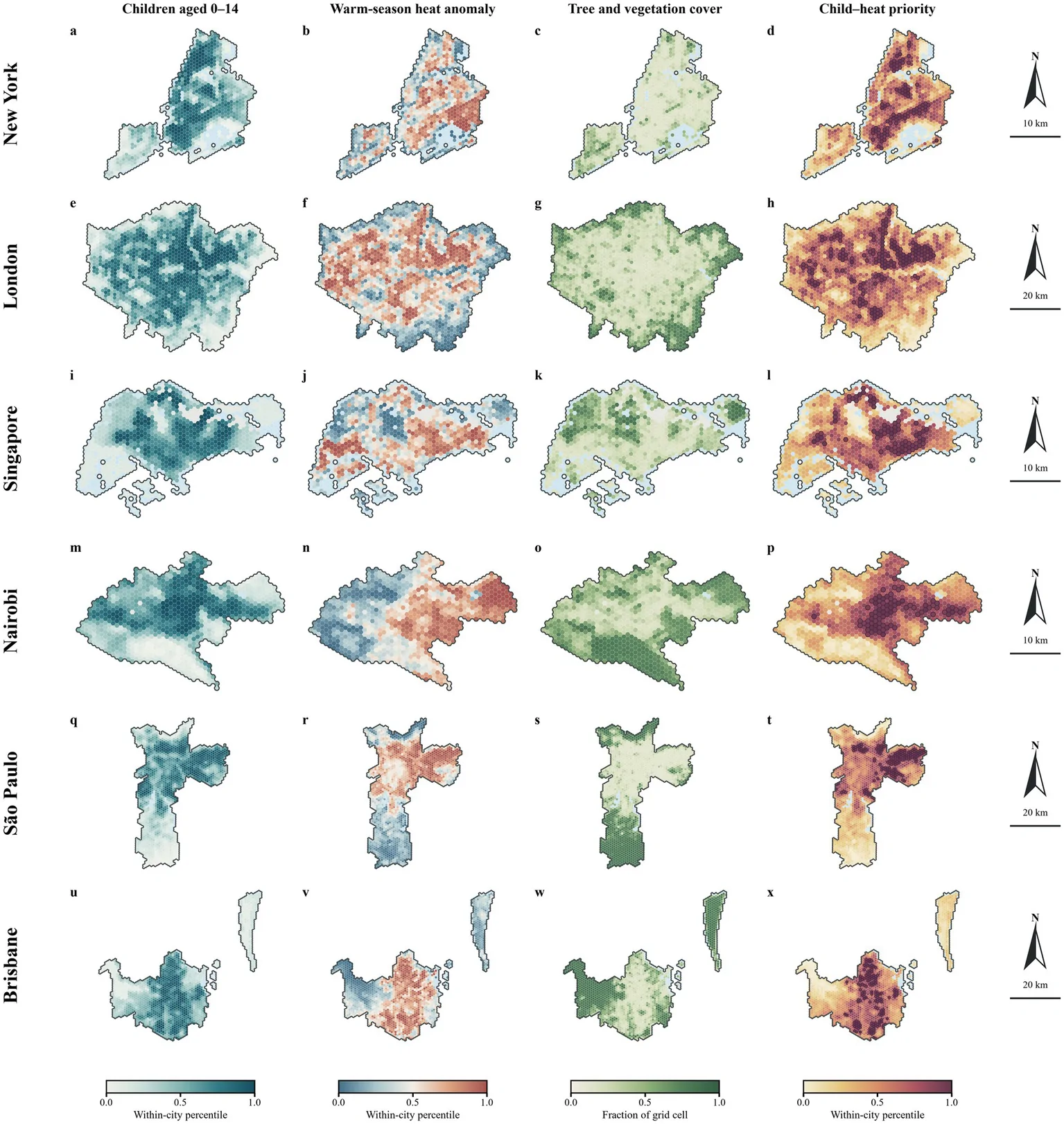
Spatial components of child–heat priority over the complete 2016–2025 period. Panels **(a–d)**, **(e–h)**, **(i–l)**, **(m–p)**, **(q–t)** and **(u–x)** represent New York, London, Singapore, Nairobi, São Paulo and Brisbane, respectively. Within each four-panel city group, the panels show, from left to right, children aged 0–14, warm-season city-year heat anomaly, combined tree and vegetation cover, and child–heat priority. Priority is the geometric mean of the child and heat percentiles only; land cover is a planning-context layer and is not included in the score.

### 3.3 Spatial distribution, annual recurrence and period-composite persistence

Priority ranks changed in both directions within every city. Positive and negative percentile-point changes occurred during the early-to-middle and middle-to-recent transitions, rather than forming a uniform increase or decrease across the administrative area (Figure 4). The citywide median change remained close to zero because ranks were recalculated within each city-period, but cell-level movement was appreciable. Singapore had the largest mean absolute change in both steps, reaching 9.60 percentile points from early to middle and 8.75 points from middle to recent. London and Nairobi showed the next largest redistributions. Thus, a stable citywide median did not imply a stationary priority geography.

**Figure 4:**
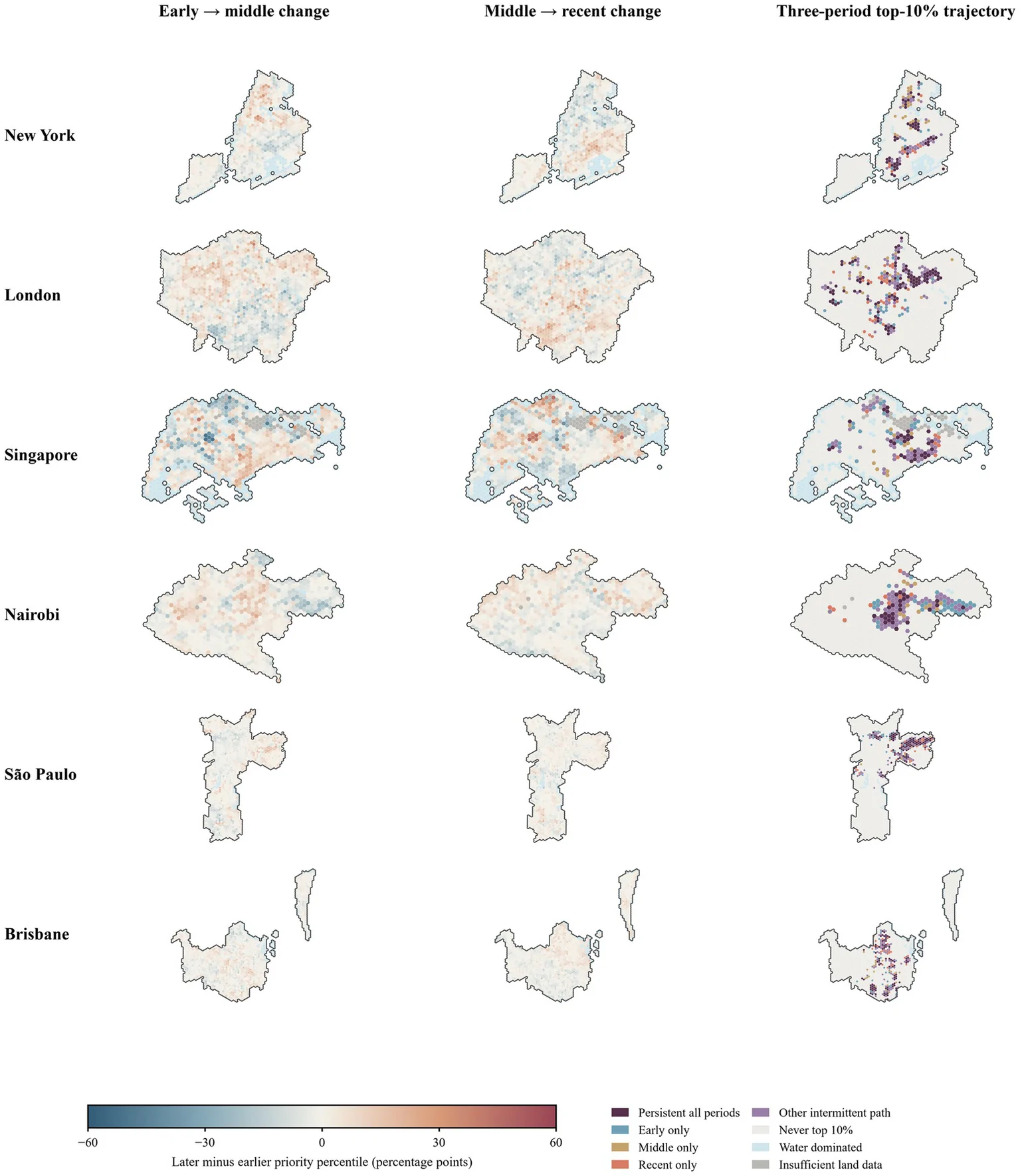
Spatial change and period-composite persistence of child–heat priority across three periods. The first two columns show percentile-point changes from early to middle and from middle to recent periods; the third column classifies upper-decile membership across all three periods. Pale blue denotes water-dominated cells and grey denotes non-priority or ineligible land, as applicable.

Period-composite persistent upper-decile areas coexisted with newly selected, declining and intermittent areas. Period-composite persistent cells numbered 102 in São Paulo, 96 in London, 84 in Brisbane, 49 in New York, 28 in Singapore and 26 in Nairobi, but these absolute counts reflected different eligible city sizes. After division by non-water land cells eligible in all three periods, period-composite persistence was 6.12% in São Paulo, 5.76% in New York, 5.66% in Brisbane, 5.57% in London, 3.85% in Singapore and 3.20% in Nairobi. Relative to cells that entered the upper decile in at least one period, the corresponding proportion ranged from 19.3% in Nairobi to 44.7% in São Paulo. Singapore combined high child concentration with low standardised period-composite persistence, demonstrating that the number of children captured and the durability of the selected geography remained distinct.

Annual metrics qualified this three-period picture. Among 10-year common-panel cells, locations classified as period-composite persistent had median annual upper-decile membership of 0.8–0.9, compared with 0.0 among other cells. The Spearman association between the period-composite indicator and annual membership proportion ranged from 0.40 to 0.63. When annual persistence was defined as membership in at least eight of 10 years, its Jaccard agreement with period-composite persistence ranged from 0.55 in Nairobi and Singapore to 0.71 in Brisbane (κ = 0.69–0.82). Requiring annual membership in all 10 years produced much smaller sets and Jaccard values of 0.08–0.29. Period-composite persistence therefore identified recurrent priority geographies but did not establish uninterrupted annual membership (Supplementary Tables S14, S15).

The proportion of children located in the upper-decile cells remained high throughout the decade, but the level and variability differed among cities (Figure 5a). Singapore had the highest 10-year mean concentration and ranged from 35.2 to 51.6%. Brisbane remained within a narrow high range of 33.2–35.8%, whereas Nairobi varied from 21.4 to 38.8%. New York showed a more moderate range of 26.1–30.5%, and London and São Paulo remained within narrower lower bands. Ten-year means therefore described the overall level of concentration, while annual ranges distinguished stable from variable cases (Figure 5b). Period-composite persistence did not follow the same cross-city ordering: São Paulo had the largest standardised period-composite persistent share, whereas Singapore and Nairobi had the smallest (Figure 5c; Supplementary Table S5). Accordingly, the share of children captured annually and persistence across period composites described complementary properties of the pattern.

**Figure 5:**
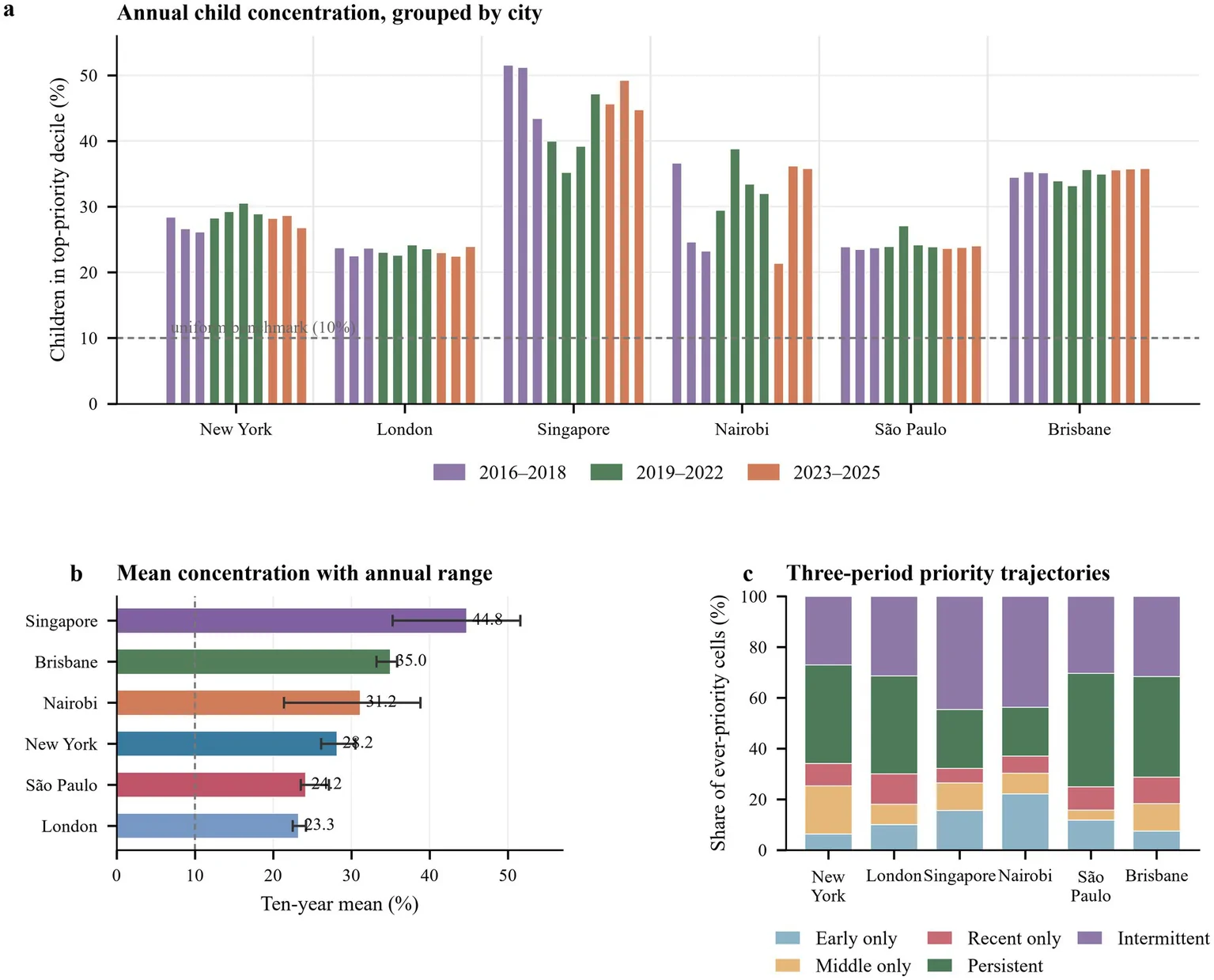
Ten-year child–heat priority dynamics across the six cities. **(a)** Annual percentage of eligible children located in upper-decile priority cells, with bars coloured purple for 2016–2018, green for 2019–2022 and orange for 2023–2025; the dashed line marks the 10% uniform benchmark. **(b)** Ten-year mean child concentration and annual range for each city. **(c)** Proportions of ever-priority cells classified as early-only, middle-only, recent-only, period-composite persistent or intermittent in each city.

### 3.4 Cross-sensor agreement, land-cover gradients and robustness analyses

Landsat and season-matched MODIS/Terra produced positively associated heat rankings in all six cities, although the strength of cross-product agreement varied (Figures 6a,b). Pooled Spearman correlations were 0.35 in New York, 0.54 in London, 0.58 in Singapore, 0.86 in Nairobi, 0.64 in São Paulo and 0.84 in Brisbane. The 5-km spatial cluster-bootstrap intervals retained the same broad ordering when all years within sampled blocks were carried together. City-year estimates provided a complementary temporal check and are reported in Supplementary Table S13 rather than treating pooled grid-year observations as temporally independent.

**Figure 6:**
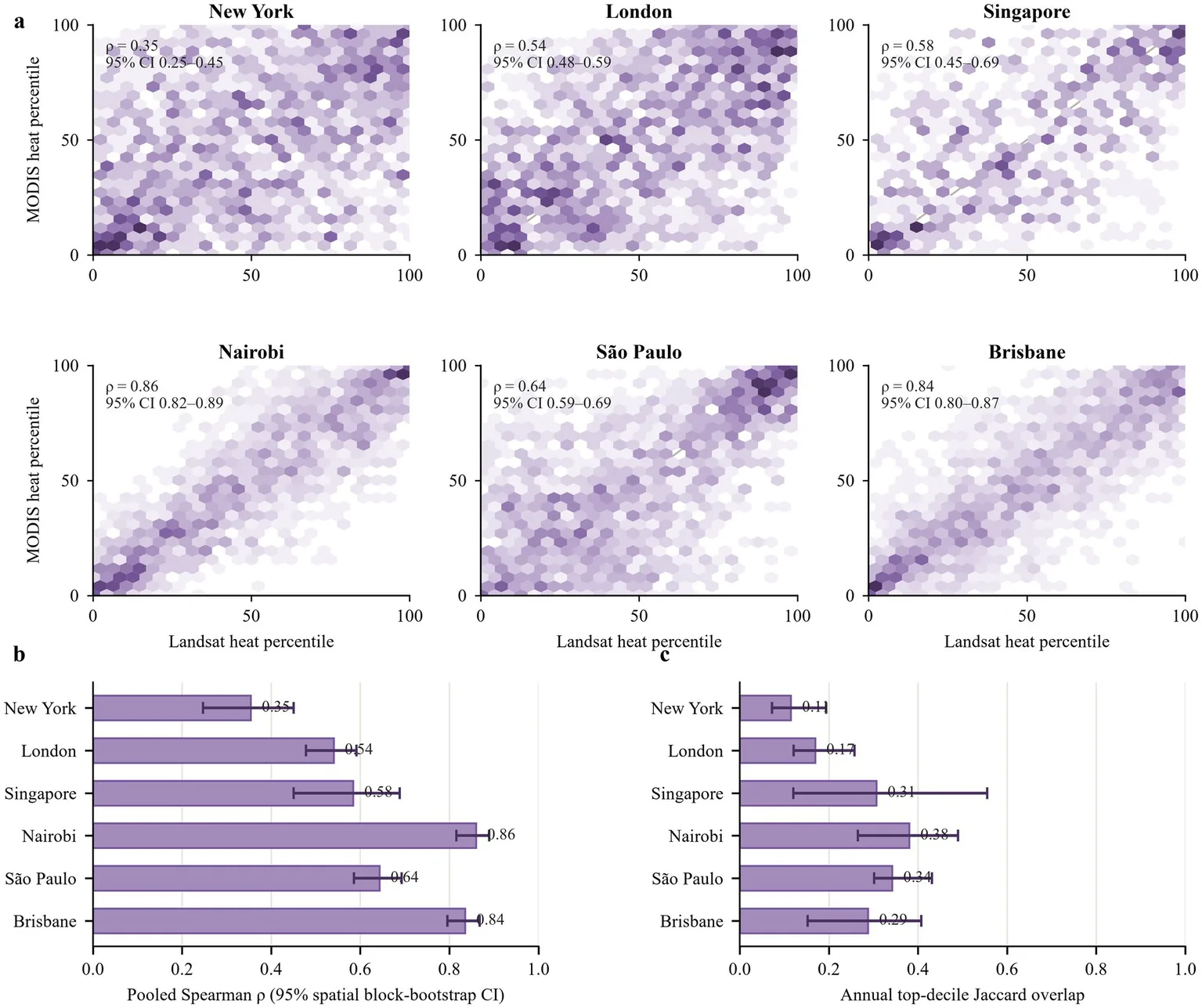
Cross-sensor agreement between Landsat and MODIS/Terra thermal rankings. **(a)** Hexagonal-bin distributions of Landsat and season-matched MODIS heat percentiles on the 0–100 scale for New York, London, Singapore, Nairobi, São Paulo and Brisbane. **(b)** Pooled Spearman correlations with 95% confidence intervals obtained from 1,000 resamples of 5-km spatial blocks. **(c)** Median annual upper-decile Jaccard overlap with the corresponding annual range for each city.

Agreement declined when the comparison was restricted to exact upper-decile membership. Median annual Jaccard overlap ranged from 0.11 to 0.38 and was lower than the corresponding full-distribution rank correlations (Figure 6c). Strong agreement in the relative ordering of all cells therefore did not guarantee that Landsat and MODIS selected identical cells around the upper-decile boundary. This pattern represents cross-product boundary sensitivity rather than failure of either sensor against a definitive reference product.

Combined tree and vegetation cover was negatively associated with the annual heat-anomaly percentile in five cities, with correlations of −0.82 in São Paulo, −0.66 in London, −0.52 in Brisbane, −0.35 in Nairobi and −0.26 in New York (Figure 7). Singapore showed a near-zero association (*ρ* = 0.03). London and São Paulo showed comparatively clear declining gradients, whereas the binned medians were weaker or less regular in Singapore and Nairobi. Greater vegetation cover was therefore generally associated with a lower within-city thermal rank, but the relationship was strongly context dependent and cannot be represented by a universal cross-city greening coefficient. The implications of unmeasured urban form, irrigation, topography and socioeconomic confounding are considered in the Discussion. Across all eligible grid-years, the pooled Spearman association between combined tree and vegetation cover and annual heat-anomaly percentile was −0.494. In leave-one-city-out analyses, the pooled association remained negative after every omission and ranged from −0.551 when New York was omitted to −0.375 when São Paulo was omitted, indicating that the overall land-cover pattern was not attributable to a single city (Supplementary Table S21).

**Figure 7:**
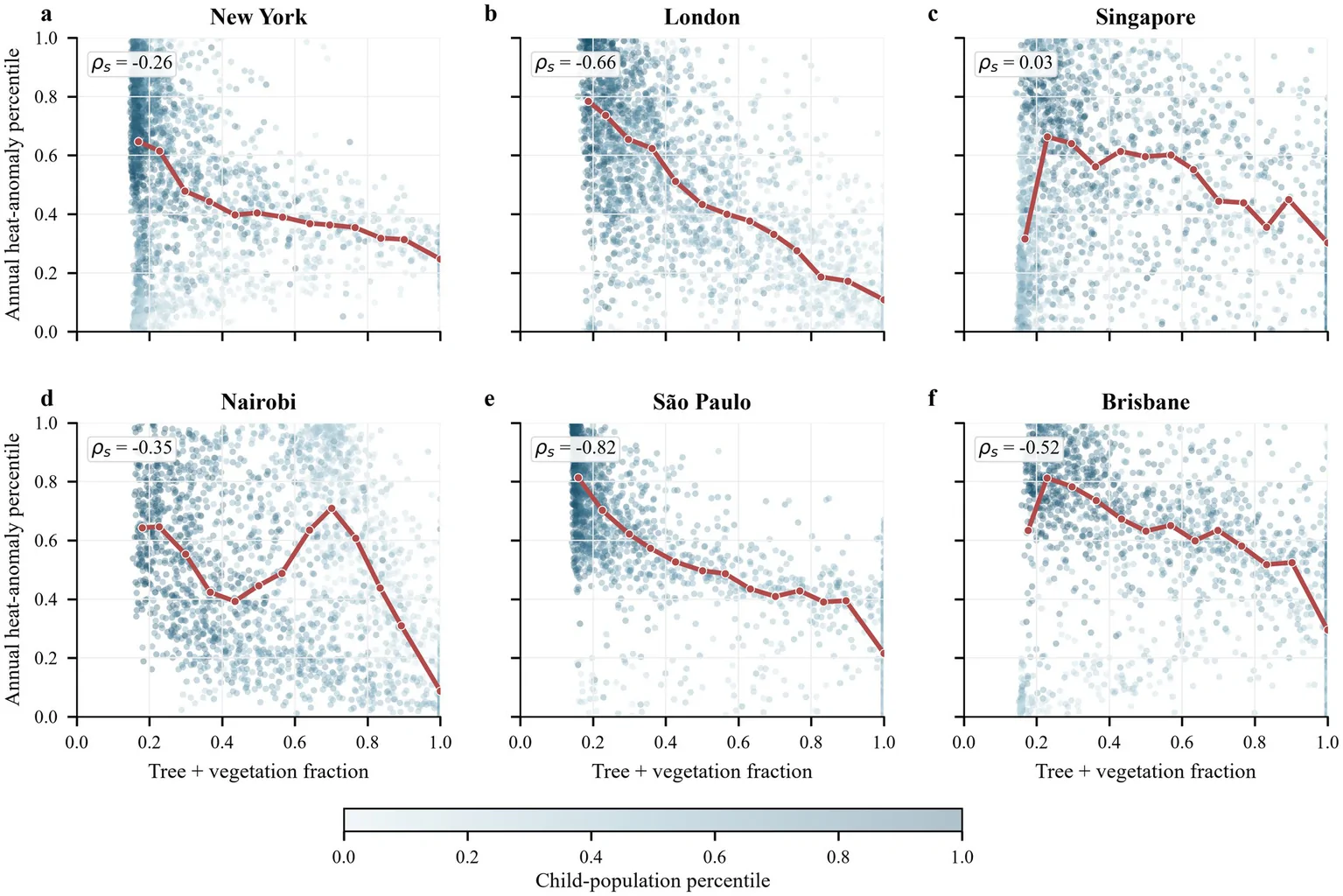
City-specific relationships between vegetation cover and heat-anomaly percentile. Panels **(a–f)** represent New York, London, Singapore, Nairobi, São Paulo and Brisbane, respectively. Points are grid-year observations; the x-axis represents combined tree and vegetation fraction, the y-axis represents the city-year heat-anomaly percentile, and point colour represents the child-population percentile. Red curves connect binned median heat percentiles. Each panel reports the Spearman correlation calculated from all complete grid-years.

Several alternative specifications were analytically equivalent to the primary index. Population share produced the same within-city-year ranks as child count because its denominator was constant within each city-year. Clipped-cell density also produced nearly identical annual priority sets (median Jaccard = 1.000; minimum = 0.988). Raw LST and the within-city heat anomaly yielded identical ranks because subtracting a common city-year median did not change their ordering. The rank/n and zero-based percentile conventions likewise selected identical annual sets. Alternative aggregation functions preserved most priority-set membership, with median Jaccard overlaps of 0.966 for both the arithmetic and harmonic means and 0.760 for the minimum-component formulation. Exact membership was more sensitive to unequal weighting: median overlap was 0.679 under child-dominant weighting (0.75/0.25) and 0.658 under heat-dominant weighting (0.25/0.75). The component weights therefore affected the resulting planning boundary and were not empirically determined by the analysis.

Changes to the selection threshold altered the proportion of children captured by the priority set. Median child concentration was 16.7%, 28.8 and 49.4% under the upper 5%, 10 and 20% thresholds, respectively. Increasing the valid-pixel coverage requirement to 70% retained a median Jaccard overlap of 1.000, with a minimum of 0.789. After restriction to 10-year common spatial support, overlap ranged from 0.84 in Brisbane to 1.00 in New York and Nairobi. Leave-one-year-out analyses likewise indicated generally stable priority-set membership: city-level median Jaccard overlap ranged from 0.822 in Nairobi to 0.920 in São Paulo, while the minimum overlap across omitted years ranged from 0.739 in Singapore to 0.891 in New York (Supplementary Table S12). The climatological assessment supported the primary windows in all six cities. For Singapore, April–June was adopted as the primary window because it spans the annual temperature peak, while March–May was retained as the comparator. The two windows preserved the continuous ordering of priority scores (median *ρ* = 0.952), but agreement between the annual upper-decile sets was moderate (median Jaccard = 0.621). Broad spatial ordering was therefore more stable than exact threshold-defined membership under a one-month seasonal shift. Full results are reported in Supplementary Tables S8–S12, S16–S19.

## 4 Discussion

The central empirical finding is not simply that high surface-heat priority areas occurred in all six cities. Four planning judgements diverged: how many children were contained in the annual upper decile, how often cells entered that decile annually, whether they remained selected across three period composites, and whether an independent thermal product reproduced the same boundary. The population-informed sets contained more children than heat-only sets in every city-year, yet cities with the greatest concentration did not have the largest standardised period-composite persistent share. Annual recurrence and period-composite persistence were related but not interchangeable, and Landsat and MODIS agreed more strongly on continuous thermal ranks than on upper-decile membership. The framework therefore separates evidence dimensions that a single hotspot map would conflate.

### 4.1 Positioning within literature

The concentration of children within a small spatial fraction extends previous work on unequal urban thermal conditions. Long-term research in the Shandong Peninsula showed that population distributions and heat risk can evolve jointly (16). On a common intra-urban grid, the present joint index placed 23.3–44.8% of eligible children in its upper decile on average, compared with 7.4–21.1% under heat-only selection. This difference is the relevant incremental result because child population is intentionally included in the joint score; concentration above a 10% spatial reference is not, by itself, an independent test of inequality. Because all components were ranked within city and year, the analysis does not rank cities by absolute temperature, morbidity or individual risk.

The more consequential contribution is the separation of concentration, annual recurrence and period-composite persistence. Singapore had the highest mean child concentration but only 3.85% of its common eligible land cells were period-composite persistent, whereas São Paulo had a lower mean concentration but the highest standardised period-composite share at 6.12%. Period-composite persistent cells generally entered the annual upper decile in eight or nine of 10 eligible years, yet exact annual persistence was uncommon. Concentration asks how many children fall inside the selected set in a given year; annual recurrence asks how often a location re-enters it; period-composite persistence asks whether smoothed multi-year spatial structure remains selected. Treating these as distinct outcomes avoids assigning a stronger temporal meaning to period composites than the annual data support.

The observed mixture of stable and changing priority areas is consistent with evidence that urban heat risk can follow different temporal trajectories within the same metropolitan region. Long-term mapping in the Beijing–Tianjin–Hebei urban agglomeration identified marked spatial divergence rather than uniform change in heat risk (17). Similarly, the present percentile-change maps contained simultaneous increases and decreases within every city, even though citywide median percentile change was close to zero. This apparent contrast arises because within-city percentiles describe redistribution: some cells rise in relative priority only as others fall. Mean absolute change and trajectory class are therefore more informative for local spatial reorganisation than a citywide median of reranked values.

A third contribution concerns boundary reproducibility. Positive Landsat–MODIS Spearman correlations supported the broad ordering from cooler to hotter cells, but lower Jaccard overlap showed that the exact upper-decile membership was less stable across products. The two statistics answer different questions: rank correlation evaluates the full thermal distribution, whereas Jaccard overlap evaluates a narrow planning set and changes when cells cross the threshold. MODIS was therefore treated as an independent product rather than as ground truth. The supported conclusion is that continuous thermal structure showed cross-product agreement while the precise decision boundary retained material uncertainty.

### 4.2 Urban-climate interpretation and planning implications

The association between tree and vegetation cover and heat rank differed markedly among cities. It was negative in five cities, from −0.26 in New York to −0.82 in São Paulo, but near zero in Singapore (ρ = 0.03). Reviews have shown that vegetation effects depend on climatic region, scale, vegetation type and the thermal metric used (4). Urban heat-health research likewise identifies land cover and the built environment as important modifiers of enhanced exposure, while emphasizing that their importance varies across social and climatic settings (19). A single pooled coefficient would therefore obscure the local conditions under which additional vegetation coincided with lower heat rank (4, 19).

These city-specific gradients have direct implications for interpretation, but not for causal attribution. Vegetation was analysed outside the priority score, so the observed association was not created mechanically by the index. Nevertheless, land cover and surface temperature share physical pathways, and the analysis did not estimate the temperature change caused by a specified intervention. The result should consequently be used to identify where greening, shade or surface-material assessment may be relevant, not to predict the cooling benefit of planting a particular amount of vegetation. Research on spatial inequality in green infrastructure similarly shows that distribution and accessibility matter in addition to total green quantity (20).

Period-composite persistent, recent-only and intermittent priority areas raise different planning questions rather than prescribing different interventions. Period-composite persistent cells identify locations where repeated high joint ranking may justify sustained local assessment of schools, childcare facilities, housing, shade and heat-safe walking routes. Recent-only cells warrant investigation of whether their emergence reflects demographic redistribution, changing surface conditions or altered observation coverage. Early-only cells cannot be interpreted automatically as successful adaptation because falling relative rank may result from changes elsewhere in the city. Intermittent cells remain monitoring and verification zones, particularly where categorical membership fluctuates near the upper-decile boundary. Decisions about investment require local facility, exposure and implementation evidence beyond these trajectory classes.

The child-focused interpretation is supported by evidence that heat affects paediatric health, but the present analysis should not be read as an epidemiological risk model. Systematic reviews associate high temperature and heatwaves with multiple adverse outcomes in children and emphasize variation by outcome, exposure definition and local context (1–3). Small-area studies have also reported spatial variation in paediatric acute-care responses to extreme heat and modification by neighbourhood conditions (14, 15). Those studies use health outcomes to estimate exposure–response relationships. The current framework instead identifies places where larger child populations and higher relative surface heat coincide, providing a first-stage spatial screen for cities in which comparable health records are unavailable.

This distinction changes how the maps should be used. A high-ranked cell is a candidate for local investigation, not evidence that an individual child experienced a particular temperature or health outcome. Facility locations, building condition, indoor overheating, air-conditioning access, travel behaviour, humidity, air pollution and community priorities can all modify the practical significance of the mapped score. Local planners should therefore combine period-composite persistent and emerging priority cells with school and childcare inventories, pedestrian networks, building audits and locally observed air temperature before selecting an intervention. The contribution of the framework is to organise where such detailed assessment begins, not to prescribe a universal response.

### 4.3 Methodological contribution and uncertainty

The methodological contribution lies in joining annual temporal alignment, common spatial support, explicit missing-data rules and independent thermal comparison in one reproducible chain. Many heat-vulnerability applications combine indicators drawn from different years or spatial units, which can convert temporal mismatch into apparent spatial inequality (7, 8). Here, annual child population and thermal observations were aligned from 2016 to 2025 on the same 1-km hexagonal support, and land-cover fractions were retained as a contextual layer. Period composites were calculated only from eligible annual observations, and missing land cells were distinguished from low-ranked land and water. This design makes the resulting transitions auditable rather than treating unavailable observations as environmental improvement.

The geometric mean serves a transparent analytical purpose by limiting compensation between the child and heat components, but it does not constitute a uniquely validated planning utility function. Population count reflects the number of children potentially served by place-based investment, whereas density would prioritise spatial intensity; equal weighting gives the two dimensions equal normative importance. Sensitivity results showed that count, population share, clipped-cell density and raw-versus-relative heat ranking made little difference to the annual sets, while unequal component weights changed exact membership appreciably. The primary score should therefore be interpreted as a reproducible reference specification whose weights can be adapted through local deliberation, not as an empirically optimal or universally transferable index.

The paired Landsat–MODIS comparison demonstrates why uncertainty should accompany threshold-based screening. Continuous rank agreement supported the broad spatial ordering of heat, whereas lower upper-decile overlap identified an uncertainty zone around the policy cut-off. The top 10% should therefore not be treated as a hard physical boundary. Cells just below and above the threshold may be practically similar, and their classification can vary with sensor resolution, cloud-free sampling and annual coverage. Reporting the continuous score together with trajectory class and cross-product agreement statistics provides a more defensible basis for screening than presenting a binary hotspot map alone.

### 4.4 Limitations and research needs

First, Landsat LST measures surface thermal conditions at satellite overpass rather than the air temperature, humidity, nighttime heat, frequency or duration of extreme heat that shape physiological stress. Warm-season median LST also does not measure time spent at home, school or outdoors, indoor overheating, mobility, protective behaviour or personal exposure. The maps should therefore be interpreted as ecological screening of relative surface-heat context, not as measurements of children’s actual heat exposure.

Second, WorldPop is a modelled redistribution of population rather than a direct enumeration of children in each grid. Its allocation procedure provides internationally consistent high-resolution estimates but inherits uncertainty from census inputs, covariates and disaggregation (21). Aggregation across ages 0–14 further obscures differences in thermoregulation, dependence on caregivers, mobility and activity patterns between infants, younger children and early adolescents. Analyses using narrower age bands would provide a more differentiated account of child vulnerability where age-resolved population, exposure and health data can be aligned at comparable spatial and temporal resolutions. Dynamic World classifications likewise contain land-cover uncertainty, and the southern-hemisphere calendar-year land-cover window was not identical to the cross-year Landsat warm season. Land cover was therefore treated as descriptive context rather than an exactly season-matched causal exposure or a component of the priority score.

Third, within-city ranking, fixed seasonal windows, period partitions and the 10% selection threshold are analytical conventions. Percentiles improve comparison of local spatial position but suppress absolute climatic and population differences. The 5%, 10 and 20% thresholds and alternative score formulations did not alter the qualitative distinction among concentration, annual recurrence and period-composite persistence, although unequal component weights, the one-month shift in Singapore’s seasonal window and adjacent period boundaries affected exact priority-set membership. Observation density also varied across city-years. The 2020 São Paulo composite retained substantial spatial coverage and satisfied the study’s stated eligibility criteria. However, its comparatively sparse within-year observations increase uncertainty around the absolute LST estimate, which should therefore not be interpreted as evidence of a climatic trend. The persistence of the low median under fixed common spatial support indicates that changing grid composition was not its sole explanation. Singapore likewise did not provide a stable all-year footprint under every restriction. Leave-one-city-out analyses showed that the pooled land-cover–heat association remained negative after each city was omitted, although its magnitude was attenuated when São Paulo was excluded. Leave-one-year-out and 10-year common-support analyses additionally evaluated temporal and spatial-sample stability. Together, these analyses reduced, but could not eliminate, dependence on the composition of the city sample and satellite availability. The six purposively selected cities remain contrasting cases rather than a representative global sample.

Fourth, administrative boundaries do not necessarily coincide with functional urban regions, commuting areas or the full footprint of urban heat processes. Coastal morphology and water exclusion were particularly influential in New York and Singapore. Boundary sensitivity using functional urban areas would help determine whether the identified patterns persist under alternative spatial frames. Most importantly, no individual medical records, school absence data or intervention outcomes were included. The study cannot estimate morbidity, causal mechanisms or adaptation effectiveness.

Future research should proceed in three directions. The first is outcome validation through spatially and temporally compatible paediatric emergency, hospital or school-absence records where governance and privacy conditions permit. The second is exposure refinement using air temperature, humidity, building and mobility data to connect residential screening with the environments children actually occupy. The third is intervention evaluation: period-composite persistent and emerging priority cells can define candidate locations for quasi-experimental assessment of greening, shade, cool materials or heat-safe school routes. These extensions would transform the present first-stage screen into a broader urban-climate and child-health decision framework.

## 5 Conclusion

A harmonised 2016–2025 panel showed that the questions most relevant to spatial screening—where children were concentrated, how often cells entered annual priority sets, which areas remained selected in period composites and whether exact boundaries were independently reproducible—did not yield the same city ordering or spatial answer. The annual upper decile contained substantially more than 10% of eligible children in every city-year, but cities with greater child concentration did not necessarily have more recurrent or period-composite persistent priority locations. The moderate rather than complete agreement between annual and period-composite measures is the principal temporal contribution over a single-year hotspot map.

The spatial analysis identified period-composite persistent, emerging, declining and intermittent priority cells, while cross-sensor comparison showed that continuous Landsat and MODIS thermal ranks were more consistent than their exact upper-decile hotspot sets. Vegetation was negatively associated with heat rank in five cities but showed a near-zero relationship in Singapore, reinforcing the context dependence of land-cover–heat gradients. These findings argue against relying on a single-year hotspot map, a universal vegetation coefficient or an unqualified binary threshold.

Three practical conclusions follow. First, period-composite persistent and recent-only cells identify different candidates for local assessment, but trajectory class alone does not establish which intervention should be funded. Second, continuous priority scores should accompany any upper-decile map because categorical membership is less stable than the underlying thermal ranking. Third, vegetation planning should be informed by city-specific land-cover–heat relationships and child distributions rather than by transferring a single canopy target between climates. These conclusions organise subsequent assessment and monitoring; they do not demonstrate the effectiveness of a specific intervention.

The framework is intended for first-stage urban adaptation screening. It provides a reproducible way to locate areas where child population and relative surface heat coincide, quantify annual recurrence, distinguish period-composite continuity from changing priority, and identify where local evidence should be collected next. It does not estimate individual exposure, clinical risk or causal intervention effects. Used with school locations, building conditions, air-temperature observations and community knowledge, the approach can support more targeted and uncertainty-aware child-focused heat adaptation in cities where comparable health records remain unavailable.

The next analytical step is external validation in cities where paediatric health, school-absence or facility-level exposure data can be linked under appropriate governance. Replication with functional urban boundaries, air-temperature and humidity observations, and alternative spatial resolutions would further test the stability of the cross-city findings. The present analysis supplies the reproducible spatial and temporal foundation for those extensions.

## Data Availability

The datasets presented in this study can be found in online repositories. The names of the repository/repositories and accession number(s) can be found in the article/Supplementary material.
