# Supplementary material for: Annual recurrence and period-composite persistence of child–heat priority across six contrasting urban systems
Source: Front Public Health. 2026 Oct 20;14:1984653. doi: 10.3389/fpubh.2026.1984653 (PMC13727534; doi:10.3389/fpubh.2026.1984653)
Supplement: Supplementary file 1 [file Data_Sheet_1.docx]

Supplementary Material

# 1 Supplementary Methods S1. Evidence searches and screening

PubMed, Scopus and Web of Science Core Collection exports were combined for a PRISMA-guided evidence-mapping exercise (1). The exports contained 1,728 records (555 from PubMed, 794 from Scopus and 379 from Web of Science). After 711 duplicates were removed, 1,017 titles and abstracts were screened. Sixty-nine reports were sought for retrieval, 62 were assessed for eligibility and 42 records (33 empirical studies and nine reviews) were retained in the structured evidence map.

## 1.1 Supplementary Fig. S1. PRISMA-guided evidence selection.

*Note. The database exports were re-screened using the child–heat and urban-spatial eligibility criteria applied in this study. Because the exports did not retain complete platform search histories, Table S1 reports transparent database-specific reconstructions rather than verbatim historical queries.*

## 1.2 Supplementary Table S1. Search blocks and database-specific query reconstructions used for evidence mapping.

| **Database/block** | **Search terms or reconstruction** |
| --- | --- |
| Population | child* OR children OR adolescen* OR pediatric OR paediatric OR youth OR infant* |
| Heat exposure | heat exposure OR extreme heat OR heatwave* OR high temperature OR ambient temperature OR urban heat island OR land surface temperature |
| Urban/spatial context | urban OR city OR cities OR neighbourhood OR neighborhood OR spatial OR GIS OR built environment OR land cover OR vegetation OR green space OR environmental justice OR inequality |
| PubMed reconstruction | ((Child[MeSH] OR Infant[MeSH] OR Adolescent[MeSH] OR child*[Title/Abstract] OR adolescen*[Title/Abstract] OR pediatric[Title/Abstract] OR paediatric[Title/Abstract]) AND (Heat[MeSH] OR Hot Temperature[MeSH] OR heat*[Title/Abstract] OR heatwave*[Title/Abstract] OR "ambient temperature"[Title/Abstract] OR "urban heat"[Title/Abstract] OR "land surface temperature"[Title/Abstract])) |
| Scopus reconstruction | TITLE-ABS-KEY((child* OR adolescen* OR pediatric OR paediatric OR youth OR infant*) AND ("heat exposure" OR "extreme heat" OR heatwave* OR "ambient temperature" OR "urban heat island" OR "land surface temperature")) |
| Web of Science reconstruction | TS=((child* OR adolescen* OR pediatric OR paediatric OR youth OR infant*) AND ("heat exposure" OR "extreme heat" OR heatwave* OR "ambient temperature" OR "urban heat island" OR "land surface temperature")) |
| Audit note | The export files do not retain complete platform search-history strings. The database-specific strings above transparently reconstruct the broad population and heat searches used for the present re-screening; urban-spatial relevance was applied during eligibility assessment. The strings are not represented as verbatim historical queries. |

*Note. The search mapped methodological approaches relevant to children, heat exposure and urban-spatial analysis. It was not designed to estimate a pooled health-effect size.*

# 2 Supplementary Methods S2. Data products and temporal alignment

The grid–year panel matched annual WorldPop Global2 R2025A estimates for ages 0–14, warm-season Landsat Collection 2 Level-2 surface temperature and Dynamic World annual land-cover fractions from 2016 to 2025. MODIS/Terra MOD11A1 V061 observations were restricted to the same city-specific windows and used for independent cross-product rank comparison. All variables were aggregated to the same nominal 1-km hexagonal support before annual within-city ranking. The primary fixed windows, their climatological basis and the Singapore March–May sensitivity analysis are reported in Supplementary Tables S17–S19.

## 2.1 Supplementary Table S2. Public data products, temporal coverage and analytical roles.

| **Product** | **Period** | **Native role** | **Derived use** |
| --- | --- | --- | --- |
| WorldPop Global2 R2025A | 2016–2025 | Age-specific population estimates | Children aged 0–14 per eligible grid cell |
| Landsat 8/9 Collection 2 Level 2 | 2016–2025 | Surface temperature and QA bands | Warm-season LST and anomaly percentile |
| Dynamic World V1 | 2016–2025 | Land-cover probabilities | Annual built, tree, vegetation, bare and water fractions |
| MODIS/Terra MOD11A1 V061 | 2016–2025 | Daily 1-km daytime LST | Season-matched comparison percentiles |

*Note. Dynamic World was used only to interpret planning context and was excluded from the two-component child–heat priority score. MODIS was used only for independent cross-sensor comparison.*

# 3 Supplementary Methods S3. Eligibility and quality control

Eligibility rules were applied before percentile ranking. Landsat images were filtered by spatial extent, warm-season dates, processing level and metadata cloud cover below 90%; pixel-level QA removed fill, cloud, cloud shadow, cirrus, snow and radiometric saturation. A primary annual grid-year required at least 50% valid Landsat pixel coverage, non-missing annual LST and child count, and non-water status. A city-year required at least 30 complete grids and at least 20% of its included longitudinal grid support. For continuity with the archived period-mapping pipeline, a grid contributed to a period composite when at least one year met the 20% annual heat-validity rule and child data were available. Median child count and median heat anomaly were then calculated within the three periods without interpolation.

## 3.1 Supplementary Table S3. Eligibility and quality-control rules applied before annual and period-specific ranking.

| **Level** | **Criterion** | **Threshold or rule** |
| --- | --- | --- |
| Grid geometry | City-boundary coverage | ≥25% |
| Grid geometry | Effective land coverage | ≥50% |
| Image | Metadata cloud cover | <90% |
| Pixel | QA_PIXEL / QA_RADSAT screening | Fill, cloud, shadow, cirrus, snow and saturation removed |
| Annual grid-year | Primary valid Landsat pixel coverage | ≥50% |
| Annual grid-year | Required paired variables | Non-missing child count, annual LST and anomaly |
| City-year | Complete child–heat grids | ≥30 and ≥20% of included longitudinal support |
| Period grid | Eligible thermal years | ≥1 year at ≥20% annual heat-validity |
| Water exclusion | Median water fraction within period | <50% for land ranking |

*Note. Criteria were applied sequentially. The annual image count and mean valid-observation count were retained as audit variables rather than used as additional eligibility thresholds. Water-dominated cells and ineligible non-water land cells were retained as separate map classes and were not assigned zero values.*

# 4 Supplementary Methods S4. Priority score, trajectories and uncertainty

Within each city and year, child population and annual heat anomaly were converted to average-tie percentile ranks on a 0–1 scale. Their equal-weight geometric mean was rescaled to 0–100 and reranked within the city-year. Every cell at or above the 90th score percentile defined the annual planning-priority set; average ties at the threshold were retained, so the set could marginally exceed exactly 10%. The implemented rank/n convention produced strictly positive percentiles, including for the lowest-ranked observation. Period-specific priority was recalculated after compositing and was labelled period-composite persistence when a cell entered the upper decile in all three composites. Annual persistence was assessed separately using top-decile years, eligible-year proportion, longest consecutive run, transition frequency, entry and exit counts, and mean continuous annual priority percentile.

Cross-product uncertainty was evaluated using 1,000 resamples of 5-km spatial blocks, with all years retained whenever a block was sampled. Landsat–MODIS agreement was characterised by pooled Spearman rank correlation, city-year correlations and annual upper-decile Jaccard overlap. Child concentration was compared with 10,000 random selections of an equal number of cells and with heat-only upper-decile sets. Sensitivity analyses compared 5%, 10% and 20% thresholds; count, share and density population specifications; one-based and zero-based percentile conventions; raw and median-centred heat ranks; arithmetic, harmonic, minimum-component and unequal-weight formulations; 20%, 50% and 70% coverage rules; leave-one-city-out pooled land-cover summaries; leave-one-year-out estimates; common ten-year support; Singapore April–June versus March–May; and adjacent 3–3–4 and 4–3–3 period partitions.

## 4.1 Supplementary Table S4. Evidence map of 42 records retained after eligibility assessment.

| **Citation** | **Year** | **Journal** | **DOI link** | **Study design** | **Heat source** | **Spatial scope** | **Limitation relative to present study** |
| --- | --- | --- | --- | --- | --- | --- | --- |
| Corpuz B.; Scott E.; Zaitchik B.F.; Zeger S.; Waugh D.; Balasubramanian A.; Madrigano J.; Koehler K.; Koehl R.; McCormack M. (2026) | 2026 | Geohealth | <https://doi.org/10.1029/2025gh001501> | Case-crossover | Station/  ambient | Multi-city/national | Strong for acute within-person contrasts but depends on health records and generally does not track planning-priority trajectories. |
| Edwards (2026) | 2026 | International Journal of Environmental Health Research | <https://doi.org/10.1080/09603123.2026.2694039> | Other observational/NR | NR in abstract | Single city/region or NR | Provides condition-specific or spatial evidence; design details require full-text confirmation and cross-city transfer is limited. |
| Blackburn C.; Sugg M.M.; Ryan S.; Runkle J.D. (2026) | 2026 | Discover public health | <https://doi.org/10.1186/s12982-026-01678-8> | Other observational/NR | NR in abstract | Single city/region or NR | Provides condition-specific or spatial evidence; design details require full-text confirmation and cross-city transfer is limited. |
| Oerther S.; Phillips Z.; Zhang Z.; Salas J.; Farabi S.; Otey T. (2025) | 2025 | Heliyon | <https://doi.org/10.1016/j.heliyon.2025.e42129> | Case-crossover | NR in abstract | Single city/region or NR | Strong for acute within-person contrasts, but depends on health records and generally does not track planning-priority trajectories. |
| Reed C.; Tiger K.; Cawley M.; Sugg M.M.; Runkle J.D. (2025) | 2025 | Environmental Research: Health | <https://doi.org/10.1088/2752-5309/ae1ce1> | Case-crossover | Station/ambient | Single city/region or NR | Strong for acute within-person contrasts, but depends on health records and generally does not track planning-priority trajectories. |
| Ndovu A.; Chen C.; Schwarz L.; Lasky E.; Weiser S.D.; Benmarhnia T. (2025) | 2025 | Environmental Health Perspectives | <https://doi.org/10.1289/ehp14236> | Case-crossover | NR in abstract | Single city/region or NR | Strong for acute within-person contrasts, but depends on health records and generally does not track planning-priority trajectories. |
| Lai H.; Lee J.E.; Harrington L.J.; Ahuriri-Driscoll A.; Newport C.; Bolton A.; Salter C.; Morton S.; Woodward A.; Hales S. (2024) | 2024 | International Journal of Environmental Research and Public Health | <https://doi.org/10.3390/ijerph21091236> | Time-series | Station/ambient | Single city/region or NR | Estimates population-level temporal and lagged associations, but commonly uses aggregate outcomes and coarse exposure support. |
| Sewell K.; Paul S.; De Polt K.; Sugg M.M.; Leeper R.D.; Rao D.; Runkle J.D. (2024) | 2024 | Discover Mental Health | <https://doi.org/10.1007/s44192-023-00055-0> | Other observational/NR | NR in abstract | Single city/region or NR | Provides condition-specific or spatial evidence; design details require full-text confirmation and cross-city transfer is limited. |
| Adams K.; Knuth C.S. (2024) | 2024 | Urban Climate | <https://doi.org/10.1016/j.uclim.2024.101833> | Other observational/NR | Station/ambient | Single city/region or NR | Provides condition-specific or spatial evidence; design details require full-text confirmation and cross-city transfer is limited. |
| Kim J.; Lee J.Y. (2019) | 2019 | Environmental Research | <https://doi.org/10.1016/j.envres.2019.108650> | Time-series | Station/ambient | Multi-city/national | Estimates population-level temporal and lagged associations, but commonly uses aggregate outcomes and coarse exposure support. |
| O'Lenick C.R.; Winquist A.; Chang H.H.; Kramer M.R.; Mulholland J.A.; Grundstein A.; Sarnat S.E. (2017) | 2017 | Environmental Research | <https://doi.org/10.1016/j.envres.2017.03.021> | Other observational/NR | NR in abstract | Multi-city/national | Provides condition-specific or spatial evidence; design details require full-text confirmation and cross-city transfer is limited. |
| Xu Z.; Liu Y.; Ma Z.; Li S.; Hu W.; Tong S. (2014) | 2014 | Environmental Research | <https://doi.org/10.1016/j.envres.2014.04.021> | Time-series | Satellite | Single city/region or NR | Estimates population-level temporal and lagged associations, but commonly uses aggregate outcomes and coarse exposure support. |
| Kakkad K.; Barzaga M.L.; Wallenstein S.; Azhar G.S.; Sheffield P.E. (2014) | 2014 | Journal of Environmental and Public Health | <https://doi.org/10.1155/2014/946875> | Other observational/NR | NR in abstract | Multi-city/national | Provides condition-specific or spatial evidence; design details require full-text confirmation and cross-city transfer is limited. |
| Lai K.Y.; Bauermeister S.; Sarkar C. (2026) | 2026 | npj Mental Health Research | <https://doi.org/10.1038/s44184-026-00190-w> | Systematic/scoping review | NR in abstract | Single city/region or NR | Synthesises effects and heterogeneity; does not itself provide a deployable longitudinal spatial screen. |
| Wang J.; Cortes-Ramirez J.; Gan T.; Davies J.M.; Hu W. (2024) | 2024 | Science of the Total Environment | <https://doi.org/10.1016/j.scitotenv.2024.175863> | Systematic/scoping review | NR in abstract | Single city/region or NR | Synthesises effects and heterogeneity; does not itself provide a deployable longitudinal spatial screen. |
| Oerther S.; Bultas M. (2024) | 2024 | MCN The American Journal of Maternal/Child Nursing | <https://doi.org/10.1097/nmc.0000000000001034> | Systematic/scoping review | Station/ambient | Single city/region or NR | Synthesises effects and heterogeneity; does not itself provide a deployable longitudinal spatial screen. |
| Schapiro L.H.; McShane M.A.; Marwah H.K.; Callaghan M.E.; Neudecker M.L. (2024) | 2024 | Journal of Climate Change and Health | <https://doi.org/10.1016/j.joclim.2024.100335> | Systematic/scoping review | NR in abstract | Single city/region or NR | Synthesises effects and heterogeneity; does not itself provide a deployable longitudinal spatial screen. |
| Uibel (2022) | 2022 | International Journal of Biometeorology | <https://doi.org/10.1007/s00484-022-02310-5> | Systematic/scoping review | NR in abstract | Single city/region or NR | Synthesises effects and heterogeneity; does not itself provide a deployable longitudinal spatial screen. |
| Lakhoo D.P.; Blake H.A.; Chersich M.F.; Nakstad B.; Kovats S. (2022) | 2022 | International Journal of Environmental Research and Public Health | <https://doi.org/10.3390/ijerph19159109> | Systematic/scoping review | Station/ambient | Single city/region or NR | Synthesises effects and heterogeneity; does not itself provide a deployable longitudinal spatial screen. |
| Nguyen V.T.; Doan Q.-V.; Tran N.N.; Luong L.T.M.; Chinh P.M.; Thai P.K.; Phung D.; Le H.H.T.C.; Dang T.N. (2022) | 2022 | Environmental Science and Pollution Research | <https://doi.org/10.1007/s11356-022-21064-6> | Systematic/scoping review | Satellite+Station/ambient | Single city/region or NR | Synthesises effects and heterogeneity; does not itself provide a deployable longitudinal spatial screen. |
| Xu (2018) | 2018 | International Journal of Biometeorology | <https://doi.org/10.1007/s00484-017-1455-5> | Systematic/scoping review | Station/ambient | Single city/region or NR | Synthesises effects and heterogeneity; does not itself provide a deployable longitudinal spatial screen. |
| Xu Z.; Sheffield P.E.; Su H.; Wang X.; Bi Y.; Tong S. (2014) | 2014 | International Journal of Biometeorology | <https://doi.org/10.1007/s00484-013-0655-x> | Systematic/scoping review | NR in abstract | Single city/region or NR | Synthesises effects and heterogeneity; does not itself provide a deployable longitudinal spatial screen. |
| Liang (2026) | 2026 | Environmental Research | <https://doi.org/10.1016/j.envres.2026.123736> | Other observational/NR | NR in abstract | Single city/region or NR | Provides condition-specific or spatial evidence; design details require full-text confirmation and cross-city transfer is limited. |
| Makrufardi F.; Chuang H.-C.; Lin Y.-C.; Ho K.-F.; Chung K.F.; Chadeau-Hyam M.; Rusmawatiningtyas D.; Arguni E.; Murni I.K.; Lin S.-C. (2026) | 2026 | European Journal of Pediatrics | <https://doi.org/10.1007/s00431-026-07032-z> | Other observational/NR | Station/ambient | Single city/region or NR | Provides condition-specific or spatial evidence; design details require full-text confirmation and cross-city transfer is limited. |
| Nascimento L.F.C.; César A.C.G.; Junior J.A.C. (2025) | 2025 | Ciência & Saúde Coletiva | <https://doi.org/10.1590/1413-81232025301.19972022en> | Time-series | Station/ambient | Single city/region or NR | Estimates population-level temporal and lagged associations, but commonly uses aggregate outcomes and coarse exposure support. |
| Runkle J.D.; Reed C.; Weidner K.; Rothschild J.; Chandrasekhar T.; Sugg M.M. (2025) | 2025 | PLOS Mental Health | <https://doi.org/10.1371/journal.pmen.0000444> | Other observational/NR | NR in abstract | Single city/region or NR | Provides condition-specific or spatial evidence; design details require full-text confirmation and cross-city transfer is limited. |
| Kassem H.; Lavigne E.; Weinberger K.; Brauer M. (2025) | 2025 | Environmental Health: A Global Access Science Source | <https://doi.org/10.1186/s12940-025-01153-y> | Case-crossover | NR in abstract | Single city/region or NR | Strong for acute within-person contrasts, but depends on health records and generally does not track planning-priority trajectories. |
| Phakisi (2025) | 2025 | Children | <https://doi.org/10.3390/children12121634> | Time-series | Station/ambient | Single city/region or NR | Estimates population-level temporal and lagged associations, but commonly uses aggregate outcomes and coarse exposure support. |
| Dey C.; Wu J.; Uesi J.; Sara G.; Dudley M.; Knight K.; Scott J.G.; Jay O.; Bowden M.; Perkes I.E. (2025) | 2025 | Australian & New Zealand Journal of Psychiatry | <https://doi.org/10.1177/00048674241290449> | Time-series | Station/ambient | Single city/region or NR | Estimates population-level temporal and lagged associations, but commonly uses aggregate outcomes and coarse exposure support. |
| Fan Y.; Hu J.; Qiu L.; Wu K.; Li Z.; Feng Y.; Wu Q.; Yang M.; Tao J.; Song J.; Su H.; Cheng J.; Wang X. (2024) | 2024 | Epilepsy & Behavior | <https://doi.org/10.1016/j.yebeh.2024.109992> | Case-crossover | Station/ambient | Single city/region or NR | Strong for acute within-person contrasts, but depends on health records and generally does not track planning-priority trajectories. |
| Teyton A.; Ndovu A.; Baer R.J.; Bandoli G.; Benmarhnia T. (2024) | 2024 | Environmental Research | <https://doi.org/10.1016/j.envres.2024.118299> | Case-crossover | NR in abstract | Single city/region or NR | Strong for acute within-person contrasts, but depends on health records and generally does not track planning-priority trajectories. |
| Ye (2024) | 2024 | American Journal of Respiratory and Critical Care Medicine | Not reported in source export | Other observational/NR | NR in abstract | Single city/region or NR | Provides condition-specific or spatial evidence; design details require full-text confirmation and cross-city transfer is limited. |
| Molina Gutiérrez M.Á.; Tortajada Hernández B.; Díez-Picazo Garot C.; Camacho Gil M.; Bellido Gómez M.; Campillo López F. (2024) | 2024 | Global Pediatrics | <https://doi.org/10.1016/j.gpeds.2024.100178> | Other observational/NR | Station/ambient | Single city/region or NR | Provides condition-specific or spatial evidence; design details require full-text confirmation and cross-city transfer is limited. |
| Tse S.M.; Sambahke S.; Sajedi M.; Gravel J.; Osmanlliu E. (2024) | 2024 | Air Quality, Atmosphere & Health | <https://doi.org/10.1007/s11869-024-01610-6> | Time-series | Station/ambient | Single city/region or NR | Estimates population-level temporal and lagged associations, but commonly uses aggregate outcomes and coarse exposure support. |
| Girma B.; Liu B.; Schinasi L.H.; Clougherty J.E.; Sheffield P.E. (2023) | 2023 | Environmental Research: Health | <https://doi.org/10.1088/2752-5309/ace27b> | Case-crossover | Station/ambient | Single city/region or NR | Strong for acute within-person contrasts, but depends on health records and generally does not track planning-priority trajectories. |
| Niu L.; Girma B.; Liu B.; Schinasi L.H.; Clougherty J.E.; Sheffield P. (2023) | 2023 | Epidemiology and Psychiatric Sciences | <https://doi.org/10.1017/s2045796023000161> | Case-crossover | Station/ambient | Single city/region or NR | Strong for acute within-person contrasts, but depends on health records and generally does not track planning-priority trajectories. |
| Stowell J.D.; Sun Y.; Spangler K.R.; Milando C.W.; Bernstein A.; Weinberger K.R.; Sun S.; Wellenius G.A. (2023) | 2023 | Environmental Research: Health | <https://doi.org/10.1088/2752-5309/ac78fa> | Case-crossover | Station/ambient | Multi-city/national | Strong for acute within-person contrasts, but depends on health records and generally does not track planning-priority trajectories. |
| Niu L.; Herrera M.T.; Girma B.; Liu B.; Schinasi L.; Clougherty J.E.; Sheffield P.E. (2022) | 2022 | Paediatric and Perinatal Epidemiology | <https://doi.org/10.1111/ppe.12793> | Case-crossover | Station/ambient | Single city/region or NR | Strong for acute within-person contrasts, but depends on health records and generally does not track planning-priority trajectories. |
| Fang J.; Song J.; Wu R.; Xie Y.; Xu X.; Zeng Y.; Zhu Y.; Wang T.; Yuan N.; Xu H.; Song X.; Zhang Q.; Xu B.; Huang W. (2021) | 2021 | Environmental Science and Pollution Research | <https://doi.org/10.1007/s11356-021-12817-w> | Time-series | Station/ambient | Single city/region or NR | Estimates population-level temporal and lagged associations, but commonly uses aggregate outcomes and coarse exposure support. |
| Wilk P.; Gunz A.; Maltby A.; Ravichakaravarthy T.; Clemens K.K.; Lavigne É.; Lim R.; Vicedo-Cabrera A.M. (2021) | 2021 | Paediatrics & Child Health | <https://doi.org/10.1093/pch/pxaa096> | Other observational/NR | Station/ambient | Single city/region or NR | Provides condition-specific or spatial evidence; design details require full-text confirmation and cross-city transfer is limited. |
| Sheffield P.E.; Herrera M.T.; Kinnee E.J.; Clougherty J.E. (2018) | 2018 | Public Health | <https://doi.org/10.1016/j.puhe.2018.06.004> | Case-crossover | Station/ambient | Single city/region or NR | Strong for acute within-person contrasts, but depends on health records and generally does not track planning-priority trajectories. |
| Iñiguez C.; Schifano P.; Asta F.; Michelozzi P.; Vicedo-Cabrera A.; Ballester F. (2016) | 2016 | Environmental Research | <https://doi.org/10.1016/j.envres.2016.06.007> | Time-series | Station/ambient | Multi-city/national | Estimates population-level temporal and lagged associations, but commonly uses aggregate outcomes and coarse exposure support. |

*Note. The evidence map distinguishes empirical designs, reviews, exposure sources and spatial scopes to support the gap analysis in the Introduction. Record-level classifications were based on indexed metadata, abstracts and full text where available. No meta-analysis was undertaken*

## 4.2 Supplementary Table S5. Normalised period-composite persistence across the three analysis periods.

| **City** | Period-composite persistent cells | **Common eligible land cells** | Period-composite persistent / common eligible (%) | **Ever-priority cells** | Period-composite persistent / ever-priority (%) |
| --- | --- | --- | --- | --- | --- |
| São Paulo | 102 | 1666 | 6.12 | 228 | 44.74 |
| New York | 49 | 850 | 5.76 | 126 | 38.89 |
| Brisbane | 84 | 1485 | 5.66 | 212 | 39.62 |
| London | 96 | 1723 | 5.57 | 249 | 38.55 |
| Singapore | 28 | 728 | 3.85 | 121 | 23.14 |
| Nairobi | 26 | 812 | 3.20 | 135 | 19.26 |

*Note. Values were recalculated from the archived grid-year panel using the definitions reported in Supplementary Methods S4.*

## 4.3 Supplementary Table S6. Child concentration under joint, heat-only and random-cell selection.

| **City** | **Joint set children (%)** | **Heat-only children (%)** | **Increment (percentage points)** | **Random-cell mean (%)** | **Minimum annual increment** | **Maximum annual increment** |
| --- | --- | --- | --- | --- | --- | --- |
| Brisbane | 35.00 | 19.59 | 15.42 | 10.04 | 10.52 | 22.44 |
| London | 23.29 | 17.58 | 5.72 | 10.04 | 4.35 | 7.29 |
| Nairobi | 31.17 | 7.44 | 23.73 | 10.10 | 12.49 | 30.07 |
| New York | 28.18 | 12.11 | 16.08 | 10.05 | 12.58 | 18.59 |
| São Paulo | 24.19 | 21.09 | 3.11 | 10.04 | 2.18 | 4.40 |
| Singapore | 44.76 | 13.90 | 30.86 | 10.05 | 19.18 | 38.85 |

*Note. Values were recalculated from the archived grid-year panel using the definitions reported in Supplementary Methods S4.*

## 4.4 Supplementary Table S7. City-year thermal coverage audit.

| **City** | **Year** | **Complete grids** | **Median LST (°C)** | **Median valid fraction** | **Median observations** | Contributing images |
| --- | --- | --- | --- | --- | --- | --- |
| Brisbane | 2016 | 1583 | 38.20 | 1.000 | 3.73 | 11.0 |
| Brisbane | 2017 | 1580 | 34.94 | 1.000 | 2.69 | 11.0 |
| Brisbane | 2018 | 769 | 36.57 | 0.966 | 0.99 | 8.0 |
| Brisbane | 2019 | 1154 | 37.63 | 0.998 | 1.21 | 10.0 |
| Brisbane | 2020 | 1357 | 34.61 | 1.000 | 1.59 | 11.0 |
| Brisbane | 2021 | 1583 | 34.43 | 1.000 | 4.51 | 19.0 |
| Brisbane | 2022 | 1583 | 35.56 | 1.000 | 4.08 | 21.0 |
| Brisbane | 2023 | 1579 | 36.19 | 1.000 | 3.00 | 24.0 |
| Brisbane | 2024 | 1583 | 34.06 | 1.000 | 3.63 | 20.0 |
| Brisbane | 2025 | 1583 | 35.30 | 1.000 | 3.78 | 18.0 |
| London | 2016 | 1729 | 30.56 | 1.000 | 2.67 | 9.0 |
| London | 2017 | 1730 | 31.81 | 1.000 | 5.37 | 10.0 |
| London | 2018 | 1730 | 36.83 | 1.000 | 4.54 | 9.0 |
| London | 2019 | 1719 | 36.93 | 1.000 | 2.38 | 5.0 |
| London | 2020 | 1730 | 33.94 | 1.000 | 5.37 | 9.0 |
| London | 2021 | 1729 | 31.31 | 1.000 | 2.55 | 6.0 |
| London | 2022 | 1730 | 36.80 | 1.000 | 8.21 | 19.0 |
| London | 2023 | 1730 | 29.53 | 1.000 | 5.72 | 21.0 |
| London | 2024 | 1730 | 31.94 | 1.000 | 5.37 | 12.0 |
| London | 2025 | 1730 | 35.69 | 1.000 | 5.34 | 17.0 |
| Nairobi | 2016 | 812 | 36.45 | 1.000 | 3.54 | 6.0 |
| Nairobi | 2017 | 812 | 38.94 | 1.000 | 4.42 | 6.0 |
| Nairobi | 2018 | 812 | 37.93 | 1.000 | 2.96 | 5.0 |
| Nairobi | 2019 | 812 | 40.80 | 1.000 | 2.76 | 5.0 |
| Nairobi | 2020 | 812 | 33.93 | 1.000 | 3.82 | 6.0 |
| Nairobi | 2021 | 812 | 38.45 | 1.000 | 3.99 | 5.0 |
| Nairobi | 2022 | 812 | 39.29 | 1.000 | 7.49 | 11.0 |
| Nairobi | 2023 | 812 | 41.93 | 1.000 | 6.33 | 10.0 |
| Nairobi | 2024 | 812 | 34.06 | 1.000 | 4.32 | 11.0 |
| Nairobi | 2025 | 812 | 35.30 | 1.000 | 5.86 | 11.0 |
| New York | 2016 | 1004 | 38.79 | 1.000 | 10.00 | 18.0 |
| New York | 2017 | 1004 | 34.70 | 1.000 | 5.70 | 11.0 |
| New York | 2018 | 1004 | 36.56 | 1.000 | 4.92 | 15.0 |
| New York | 2019 | 1004 | 38.57 | 1.000 | 7.08 | 14.0 |
| New York | 2020 | 1004 | 38.69 | 1.000 | 4.77 | 15.0 |
| New York | 2021 | 1004 | 36.05 | 1.000 | 5.14 | 14.0 |
| New York | 2022 | 1004 | 39.44 | 1.000 | 11.87 | 31.0 |
| New York | 2023 | 1004 | 37.69 | 1.000 | 9.48 | 26.0 |
| New York | 2024 | 1004 | 37.57 | 1.000 | 10.92 | 31.0 |
| New York | 2025 | 1004 | 36.81 | 1.000 | 12.85 | 29.0 |
| São Paulo | 2016 | 1703 | 37.34 | 1.000 | 2.00 | 8.0 |
| São Paulo | 2017 | 1700 | 35.12 | 1.000 | 2.09 | 9.0 |
| São Paulo | 2018 | 1702 | 38.70 | 1.000 | 3.00 | 8.0 |
| São Paulo | 2019 | 1661 | 34.19 | 1.000 | 2.00 | 8.0 |
| São Paulo | 2020 | 1261 | 20.62 | 1.000 | 1.07 | 8.0 |
| São Paulo | 2021 | 1703 | 36.32 | 1.000 | 6.06 | 14.0 |
| São Paulo | 2022 | 1699 | 33.80 | 1.000 | 5.15 | 15.0 |
| São Paulo | 2023 | 1703 | 35.95 | 1.000 | 5.27 | 19.0 |
| São Paulo | 2024 | 1703 | 35.31 | 1.000 | 4.23 | 19.0 |
| São Paulo | 2025 | 1702 | 37.06 | 1.000 | 3.39 | 20.0 |
| Singapore | 2016 | 886 | 38.19 | 1.000 | 1.98 | 4.0 |
| Singapore | 2017 | 897 | 33.48 | 1.000 | 1.58 | 6.0 |
| Singapore | 2018 | 946 | 38.51 | 1.000 | 2.06 | 5.0 |
| Singapore | 2019 | 884 | 32.56 | 1.000 | 1.94 | 5.0 |
| Singapore | 2020 | 862 | 32.10 | 1.000 | 1.53 | 4.0 |
| Singapore | 2021 | 866 | 37.46 | 1.000 | 1.72 | 5.0 |
| Singapore | 2022 | 940 | 31.95 | 1.000 | 2.48 | 7.0 |
| Singapore | 2023 | 945 | 35.62 | 1.000 | 4.31 | 10.0 |
| Singapore | 2024 | 916 | 35.66 | 1.000 | 2.41 | 7.0 |
| Singapore | 2025 | 936 | 35.93 | 1.000 | 3.43 | 11.0 |

*Note. Values were recalculated from the archived grid-year panel using the definitions reported in Supplementary Methods S4.*

## 4.5 Supplementary Table S8. Sensitivity analysis restricted to ten-year common spatial support.

| **City** | **Ten-year common grids** | **Mean children in priority set (%)** | **Median overlap with primary** |
| --- | --- | --- | --- |
| Brisbane | 498 | 37.87 | 0.843 |
| London | 1718 | 23.17 | 0.989 |
| Nairobi | 812 | 31.17 | 1.000 |
| New York | 1004 | 28.18 | 1.000 |
| Singapore | 664 | 43.74 | 0.909 |
| São Paulo | 1213 | 27.58 | 0.946 |

*Note. Values were recalculated from the archived grid-year panel using the definitions reported in Supplementary Methods S4.*

## 4.6 Supplementary Table S9. Sensitivity to 5%, 10% and 20% priority thresholds.

| **Selected cell fraction** | **Median children share** | **Minimum** | **Maximum** |
| --- | --- | --- | --- |
| 0.05 | 0.167 | 0.106 | 0.340 |
| 0.10 | 0.288 | 0.214 | 0.516 |
| 0.20 | 0.494 | 0.396 | 0.787 |

*Note. Values were recalculated from the archived grid-year panel using the definitions reported in Supplementary Methods S4.*

## 4.7 Supplementary Table S10. Agreement between alternative score formulations and the geometric-mean priority set.

| **Alternative score** | **Median Jaccard** | **Minimum** | **Maximum** |
| --- | --- | --- | --- |
| arithmetic | 0.966 | 0.763 | 1.000 |
| child_weighted | 0.679 | 0.378 | 0.791 |
| harmonic | 0.966 | 0.906 | 1.000 |
| heat_weighted | 0.658 | 0.414 | 0.799 |
| minimum | 0.760 | 0.592 | 0.856 |

*Note. Values were recalculated from the archived grid-year panel using the definitions reported in Supplementary Methods S4.*

## 4.8 Supplementary Table S11. Sensitivity to alternative valid-coverage thresholds.

| **Valid-coverage threshold** | **Median Jaccard** | **Minimum Jaccard** |
| --- | --- | --- |
| 0.200 | 1.000 | 1.000 |
| 0.500 | 1.000 | 1.000 |
| 0.700 | 1.000 | 0.789 |

*Note. Values were recalculated from the archived grid-year panel using the definitions reported in Supplementary Methods S4.*

## 4.9 Supplementary Table S12. Leave-one-year-out stability of priority-cell membership.

| **City** | **Median Jaccard** | **Minimum Jaccard** |
| --- | --- | --- |
| Brisbane | 0.869 | 0.818 |
| London | 0.896 | 0.870 |
| Nairobi | 0.822 | 0.783 |
| New York | 0.912 | 0.891 |
| São Paulo | 0.920 | 0.866 |
| Singapore | 0.860 | 0.739 |

*Note. Values were recalculated from the archived grid-year panel using the definitions reported in Supplementary Methods S4.*

## 4.10 Supplementary Table S13. Annual Landsat–MODIS cross-product agreement.

| **City** | **Median annual Spearman ρ** | **Minimum annual ρ** | **Maximum annual ρ** | **Median annual Jaccard** |
| --- | --- | --- | --- | --- |
| Brisbane | 0.842 | 0.718 | 0.896 | 0.288 |
| London | 0.535 | 0.365 | 0.684 | 0.169 |
| Nairobi | 0.885 | 0.705 | 0.908 | 0.380 |
| New York | 0.358 | 0.258 | 0.439 | 0.115 |
| São Paulo | 0.636 | 0.440 | 0.803 | 0.342 |
| Singapore | 0.546 | -0.218 | 0.812 | 0.307 |

*Note. Values were recalculated from the archived grid-year panel using the definitions reported in Supplementary Methods S4.*

## 4.11 Supplementary Table S14. Annual priority recurrence, run length and transitions by city.

| **City** | **10-y common cells** | **Ever annual top 10%** | **All 10 y** | **≥8 y** | **Median top-10 years*** | **Median longest run*** | **Mean transition frequency*** | **Mean entries*** | **Mean exits*** |
| --- | --- | --- | --- | --- | --- | --- | --- | --- | --- |
| Brisbane | 498 | 89 | 7 | 29 | 5.0 | 2.0 | 0.34 | 1.52 | 1.55 |
| London | 1718 | 372 | 8 | 65 | 5.0 | 2.0 | 0.41 | 1.85 | 1.84 |
| Nairobi | 812 | 192 | 3 | 19 | 4.0 | 2.0 | 0.43 | 1.93 | 1.93 |
| New York | 1004 | 217 | 14 | 49 | 4.0 | 2.0 | 0.33 | 1.47 | 1.47 |
| São Paulo | 1213 | 221 | 7 | 73 | 6.0 | 3.0 | 0.36 | 1.63 | 1.62 |
| Singapore | 664 | 141 | 6 | 34 | 5.0 | 3.0 | 0.38 | 1.75 | 1.69 |

*Note. *Summaries are among cells entering the annual upper decile at least once. Transition frequency uses consecutive eligible-year pairs; gaps reset a run and do not create a transition.*

## 4.12 Supplementary Table S15. Agreement between annual persistence and period-composite persistence.

| **City** | **Common cells** | **Period-composite persistent** | **Median annual proportion†** | **Spearman ρ** | **Annual ≥8 y** | **Jaccard ≥8 y** | **κ ≥8 y** | **Annual all 10 y** | **Jaccard all 10 y** |
| --- | --- | --- | --- | --- | --- | --- | --- | --- | --- |
| Brisbane | 427 | 29 | 0.90 | 0.61 | 29 | 0.71 | 0.82 | 7 | 0.24 |
| London | 1711 | 96 | 0.80 | 0.54 | 65 | 0.58 | 0.72 | 8 | 0.08 |
| Nairobi | 810 | 26 | 0.80 | 0.40 | 19 | 0.55 | 0.70 | 3 | 0.12 |
| New York | 850 | 49 | 0.90 | 0.52 | 49 | 0.66 | 0.78 | 14 | 0.29 |
| São Paulo | 1176 | 80 | 0.80 | 0.63 | 73 | 0.66 | 0.78 | 7 | 0.09 |
| Singapore | 474 | 25 | 0.90 | 0.47 | 34 | 0.55 | 0.69 | 6 | 0.19 |

*Note. Analyses are restricted to cells eligible in all ten annual panels and comparable across all three period composites. †Among period-composite persistent cells.*

## 4.13 Supplementary Table S16. Sensitivity to population, percentile, heat and score-construction choices.

| **Alternative specification** | **Median Jaccard** | **Minimum** | **Maximum** | **City-years** |
| --- | --- | --- | --- | --- |
| Arithmetic mean, equal weights | 0.966 | 0.763 | 1.000 | 60 |
| Child density per clipped km2 | 1.000 | 0.988 | 1.000 | 60 |
| Child-weighted geometric mean (0.75/0.25) | 0.679 | 0.378 | 0.791 | 60 |
| Harmonic mean, equal weights | 0.966 | 0.906 | 1.000 | 60 |
| Heat-weighted geometric mean (0.25/0.75) | 0.658 | 0.414 | 0.799 | 60 |
| Minimum component percentile | 0.760 | 0.592 | 0.856 | 60 |
| Population share (rank-equivalent to count) | 1.000 | 1.000 | 1.000 | 60 |
| Raw annual LST in place of anomaly | 1.000 | 1.000 | 1.000 | 60 |
| Zero-based percentiles | 1.000 | 1.000 | 1.000 | 60 |

*Note. Jaccard overlap compares each annual alternative with the primary equal-weight geometric-mean set. Population share is rank-equivalent to count within a city-year, and subtracting a common city-year LST median leaves heat ranks unchanged.*

## 4.14 Supplementary Table S17. City-specific warm-season definitions and climatological rationale.

| **City** | **Primary window** | **Rationale** | **Institutional source** | **URL** |
| --- | --- | --- | --- | --- |
| New York | June–August | Warmest contiguous three months in Central Park 1991–2020 normals | US National Weather Service, New York City climate normals | <https://www.weather.gov/media/okx/Climate/CentralPark/nycnormals.pdf> |
| London | June–August | Warmest contiguous three months in Greenwich 1991–2020 averages | UK Met Office, Greenwich long-term averages | <https://www.metoffice.gov.uk/research/climate/maps-and-data/location-specific-long-term-averages/u10hb5> |
| Singapore | April–June | Climatology-based window spanning the annual temperature peak; tied highest contiguous three-month 24-hour mean | Meteorological Service Singapore, Climate of Singapore | <https://www.weather.gov.sg/climate-climate-of-singapore/> |
| Nairobi | January–March | Broadly hottest contiguous quarter in Nairobi Dagoretti climatology | UN-Habitat report reproducing Nairobi Dagoretti climatic data | <https://unhabitat.org/sites/default/files/2020/06/gh050e_compressed.pdf> |
| São Paulo | December–February | Austral-summer warmest contiguous quarter in 1991–2020 normals | INMET, Climatological Normals of Brazil 1991–2020 | <https://portal.inmet.gov.br/normais> |
| Brisbane | December–February | Austral-summer warmest contiguous quarter in station normals | Australian Bureau of Meteorology, Brisbane Aero climate averages | <https://www.bom.gov.au/climate/averages/tables/cw_040913.shtml> |

*Note. Sources were accessed 22 September 2026. The windows standardise within-city annual sampling and do not imply meteorologically equivalent seasons across climate regimes. Singapore’s April–June primary window spans the annual temperature peak and is tied for the highest contiguous three-month mean in the official 1991–2020 24-hour temperature normals; March–May was retained as the pre-southwest-monsoon sensitivity window.*

## 4.15 Supplementary Table S18. Singapore annual sensitivity of the April–June primary window to the March–May comparator.

| **Year** | **AMJ grids** | **MAM grids** | **LST ρ** | **Priority ρ** | **Top-10 Jaccard** | **AMJ children %** | **MAM children %** | **Difference pp** |
| --- | --- | --- | --- | --- | --- | --- | --- | --- |
| 2016 | 886 | 927 | 0.938 | 0.971 | 0.640 | 51.6 | 52.2 | 0.6 |
| 2017 | 897 | 908 | 0.951 | 0.968 | 0.757 | 51.2 | 49.7 | -1.6 |
| 2018 | 946 | 946 | 0.997 | 0.998 | 1.000 | 43.4 | 43.4 | -0.0 |
| 2019 | 884 | 914 | 0.803 | 0.885 | 0.460 | 40.0 | 43.5 | 3.6 |
| 2020 | 862 | 892 | 0.768 | 0.887 | 0.639 | 35.2 | 42.5 | 7.3 |
| 2021 | 866 | 797 | 0.837 | 0.927 | 0.392 | 39.2 | 52.4 | 13.2 |
| 2022 | 940 | 905 | 0.866 | 0.933 | 0.603 | 47.2 | 45.0 | -2.2 |
| 2023 | 945 | 945 | 0.968 | 0.982 | 0.583 | 45.7 | 44.4 | -1.3 |
| 2024 | 916 | 946 | 0.905 | 0.935 | 0.571 | 49.3 | 52.7 | 3.5 |
| 2025 | 936 | 938 | 0.957 | 0.976 | 0.694 | 44.8 | 44.3 | -0.5 |

*Note. Correlations use grids complete under both windows. Difference is March–May minus April–June child concentration in percentage points.*

## 4.16 Supplementary Table S19. Singapore period-composite persistence sensitivity to the March–May comparator.

| **Comparison** | **Statistic** | **Result** |
| --- | --- | --- |
| Annual LST rank | Median Spearman ρ | 0.922 |
| Annual priority percentile | Median Spearman ρ | 0.952 |
| Annual top-decile set | Median Jaccard (range) | 0.621 (0.392-1.000) |
| Period-composite persistent set | AMJ cells / MAM cells / Jaccard | 28 / 29 / 0.425 |
| Early period top-decile set | AMJ cells / MAM cells / Jaccard | 77 / 77 / 0.656 |
| Middle period top-decile set | AMJ cells / MAM cells / Jaccard | 77 / 77 / 0.540 |
| Recent period top-decile set | AMJ cells / MAM cells / Jaccard | 77 / 77 / 0.692 |

*Note. AMJ denotes the April–June primary window and MAM denotes the March–May comparator. Continuous spatial ordering was more stable than exact top-decile boundaries under the one-month shift.*

## 4.17 Supplementary Table S20. Sensitivity to alternative three-period boundaries.

| **City** | **Period partition** | **Period-composite persistent cells** | **Jaccard with primary persistent set** |
| --- | --- | --- | --- |
| Brisbane | Primary 3-4-3 | 84 | 1.000 |
| Brisbane | Alternative 3-3-4 | 83 | 0.796 |
| Brisbane | Alternative 4-3-3 | 77 | 0.789 |
| London | Primary 3-4-3 | 96 | 1.000 |
| London | Alternative 3-3-4 | 96 | 0.829 |
| London | Alternative 4-3-3 | 99 | 0.741 |
| Nairobi | Primary 3-4-3 | 26 | 1.000 |
| Nairobi | Alternative 3-3-4 | 23 | 0.750 |
| Nairobi | Alternative 4-3-3 | 35 | 0.605 |
| New York | Primary 3-4-3 | 49 | 1.000 |
| New York | Alternative 3-3-4 | 44 | 0.755 |
| New York | Alternative 4-3-3 | 45 | 0.843 |
| São Paulo | Primary 3-4-3 | 102 | 1.000 |
| São Paulo | Alternative 3-3-4 | 103 | 0.830 |
| São Paulo | Alternative 4-3-3 | 98 | 0.739 |
| Singapore | Primary 3-4-3 | 28 | 1.000 |
| Singapore | Alternative 3-3-4 | 32 | 0.622 |
| Singapore | Alternative 4-3-3 | 29 | 0.583 |

*Note. Primary: 2016–2018 / 2019–2022 / 2023–2025; alternative 3–3–4: 2016–2018 / 2019–2021 / 2022–2025; alternative 4–3–3: 2016–2019 / 2020–2022 / 2023–2025.*

## 4.18 Supplementary Table S21. Leave-one-city-out robustness of the pooled land-cover–heat association.

| **Omitted city** | **Eligible grid-years** | **Pooled Spearman ρ** |
| --- | --- | --- |
| None (all cities) | 72,934 | −0.494 |
| Brisbane | 58,695 | −0.509 |
| London | 55,718 | −0.463 |
| Nairobi | 64,914 | −0.506 |
| New York | 62,894 | −0.551 |
| São Paulo | 56,527 | −0.375 |
| Singapore | 65,922 | −0.548 |

*Note. Spearman correlations were calculated across eligible grid-years after pooling the remaining cities. Combined tree and vegetation fraction was compared with the annual within-city heat-anomaly percentile. The association remained negative after every city omission; the weaker magnitude after omitting São Paulo indicates contextual influence without reversal of the pooled pattern.*

# References

1.	Page MJ, McKenzie JE, Bossuyt PM, Boutron I, Hoffmann TC, Mulrow CD, et al. The PRISMA 2020 statement: an updated guideline for reporting systematic reviews. *BMJ* (2021) 372. doi: [10.1136/bmj.n71](https://doi.org/10.1136/bmj.n71).
